# Controlling COVID-19 outbreaks in the correctional setting: A mathematical modelling study

**DOI:** 10.1371/journal.pone.0303062

**Published:** 2024-05-17

**Authors:** Neil Arvin Bretaña, Jisoo A. Kwon, Luke Grant, Jennifer Galouzis, Colette McGrath, Wendy Hoey, James Blogg, Andrew R. Lloyd, Richard T Gray

**Affiliations:** 1 Allied Health and Human Performance, University of South Australia, Australia; 2 Kirby Institute, UNSW Sydney, Sydney, New South Wales, Australia; 3 Corrective Services NSW, Australia; 4 Justice Health and Forensic Mental Health Network NSW, Australia; Interfaculty Center of Training and Research in Environment and Development, Universite d’Abomey-Calavi, BENIN

## Abstract

Correctional centres (termed here ‘prisons’) are at high risk of COVID-19 and have featured major outbreaks worldwide. Inevitable close contacts, frequent inmate movements, and a disproportionate burden of co-morbidities mean these environments need to be prioritised in any public health response to respiratory pathogens such as COVID-19. We developed an individual-based SARS-CoV-2 transmission model for the prison system in New South Wales, Australia ‐ incorporating all 33 correctional centres, 13,458 inmates, 578 healthcare and 6,909 custodial staff. Potential COVID-19 disease outbreaks were assessed under various mitigation strategies, including quarantine on entry, isolation of cases, rapid antigen testing of staff, as well as immunisation.Without control measures, the model projected a peak of 472 new infections daily by day 35 across the prison system, with all inmates infected by day 120. The most effective individual mitigation strategies were high immunisation coverage and prompt lockdown of centres with infected inmates which reduced outbreak size by 62–73%. Other than immunisation, the combination of quarantine of inmates at entry, isolation of proven or suspected cases, and widespread use of personal protective equipment by staff and inmates was the most effective strategy. High immunisation coverage mitigates the spread of COVID-19 within and between correctional settings but is insufficient alone. Maintaining quarantine and isolation, along with high immunisation levels, will allow correctional systems to function with a low risk of outbreaks. These results have informed public health policy for respiratory pathogens in Australian correctional systems.

## Introduction

Correctional facilities have featured several major COVID-19 outbreaks during the SARS-CoV-2 pandemic. Correctional facilities have featured several major COVID-19 outbreaks during the SARS-CoV-2 pandemic. For instance, the first case of COVID-19 recorded at a main jail complex in New York City spread to over 200 cases within the facility in the next 2 weeks [1]. A similar situation was observed at a jail in Chicago with approximately 350 cases diagnosed in April 2020 [1]. This highlights the high risk of transmission of COVID-19 and other respiratory infections, within prisons (note that the term ‘prisons’ is used here to describe correctional facilities, including gaols/jails, prisons, and other custodial settings). Inmates are particularly vulnerable due to the close living quarters, the challenges of implementation of public health control measures, and the high prevalence of underlying health conditions [2, 3]. Given this context, inmates, as well as correctional and healthcare staff, and even visitors, are at risk of infection during an outbreak in a prison system.

It is well recognised that prisons should be prioritised in the public health response to the COVID-19 pandemic, and for similar respiratory pathogens [4–7] Previous analyses of observational datasets have identified risk and mitigation factors associated with COVID-19 outbreaks in prisons. Time-series analysis on data from the California state prisons showed a positive correlation between prison transfers and COVID-19 case rates [8]. Another analysis of data from US prisons also revealed an association between the spread of COVID-19 in the community and a growing prison population [9]. These studies highlight setting-specific factors such as over-crowding and intra-system prison transfers.

Mathematical models have been widely used to inform regional and national policies and public health responses during the COVID-19 pandemic [10–15]. Previous modelling studies have quantified the potential effectiveness of individual interventions, or circumscribed sets of control measures, such as regular screening of staff to reduce this portal of viral entry, [16] decarceration or immmunisation of prisoners to reduce the size of the susceptible population, [17] as well as quarantine of all newly incarcerated individuals and use of personal protection equipment (PPE) [17]. However, these models largely lacked real world data for calibration and validation, and did not consider the differing transmissability and virulence characteristics of the SARS-CoV-2 variants of concern. In addition, these models have generally focused on a limited number of individual prisons rather than considering the whole prison system. Previous models have also disregarded the complexities of varied person-to-person interactions within a prison setting, the diverse physical structures within the prisons, and individual vulnerabilities that may influence COVID-19 infection outcomes [10, 15, 18].

For this study, we developed an individual-based mathematical model representing the prison system within the Australian state of New South Wales (NSW), the most populous state in the country. We collaborated closely in development of the model with the correctional and prison health authorities in NSW. The model incorporated data provided from the sector including inmate and staff populations, close contact rates, inmate movements, and was validated using data from outbreaks that occurred prior to immunisation scale-up. The model was then used to describe outbreak characteristics for SARS-CoV-2 strains (alpha, delta, and omicron) and to explore the efficacy of a range of integrated COVID-19 public health mitigation strategies at both the individual prison and the prison system level.

## Methods

We developed an individual-based model using C++, adapting an existing model of hepatitis C transmission, [19] to simulate SARS-CoV-2 transmission in the NSW prisons. There were 33 correctional centres in NSW at the time spread over 800,000 square Km. Eleven of these centres included facilities with more than one security classification, but with discrete boundaries, and so were considered separately. The model therefore included 27 minimum security prisons, 11 medium security prisons, and 18 maximum security prisons. The prisons included 14 ‘reception’ centres which receive newly incarcerated individuals from the community. All centres housed both individuals who have been sentenced and those not yet sentenced (i.e., on remand). Modelled individuals were inmates, correctional staff, healthcare staff, or family visitors. All individuals were assumed to be of the same gender. The model simulated daily SARS-CoV-2 transmission over 120 days, tracking individual characteristics which changed probabilistically each day (Table 1). To account for stochasticity, a total of 100 simulations were run for each scenario. Results were obtained by taking mean/median values of key indicators and a 95% confidence interval (CI) from the 100 simulations. The model code is available via an online repository under an open access license [20].

**Table 1:** Key parameters used in the model.

Parameter	Distribution	Reference
Number of individuals incarcerated per day	Uniform (*a* = 56; *b* = 62)	Corrective Services NSW
Number of correctional staff per day	Fixed value (*x* = 5932)	Corrective Services NSW
Number of healthcare staff per day	Fixed value (*x* = 578)	Corrective Services NSW
COVID-19 transmission		
alpha strain	Uniform (*a* = 0.02; *b* = 0.05)	[22]
delta strain	Uniform (*a* = 0.02; *b* = 0.05) * 2	
omicron strain	Uniform (*a* = 0.02; *b* = 0.05) * 4	
Recovery from COVID-19 infection		
asymptomatic	Lognormal (*z* = 2.07, σ = 0.24)	[22]
mild	Lognormal (*z* = 2.08, σ = 0.24)	[30]
moderate	Lognormal (*z* = 2.64, σ = 0.17)	[30]
severe	Lognormal (*z* = 3.63, σ = 0.06)	[30]
Death related to severe COVID-19 infection		
Age 19 and below	Gamma (*a* = 1.24E-4, *b* = 0.51)	[30]
Age 20 to 44	Gamma (*a* = 1.48E-2, *b* = 1.00)	[30]
Age 45 to 54	Gamma (*a* = 0.01, *b* = 0.83)	[30]
Age 55 to 64	Gamma (*a* = 0.01, *b* = 2.48)	[30]
Age 65 to 74	Gamma (*a* = 0.01, *b* = 5.34)	[30]
Age 75 to 84	Gamma (*a* = 0.01, *b* = 6.87)	[30]
Age 85 and above	Gamma (*a* = 0.02, *b* = 6.90)	[30]

Other model parameters listed in S1 to S17 Tables in S1 File.

### Population and prison system structure

**Fig 1:**
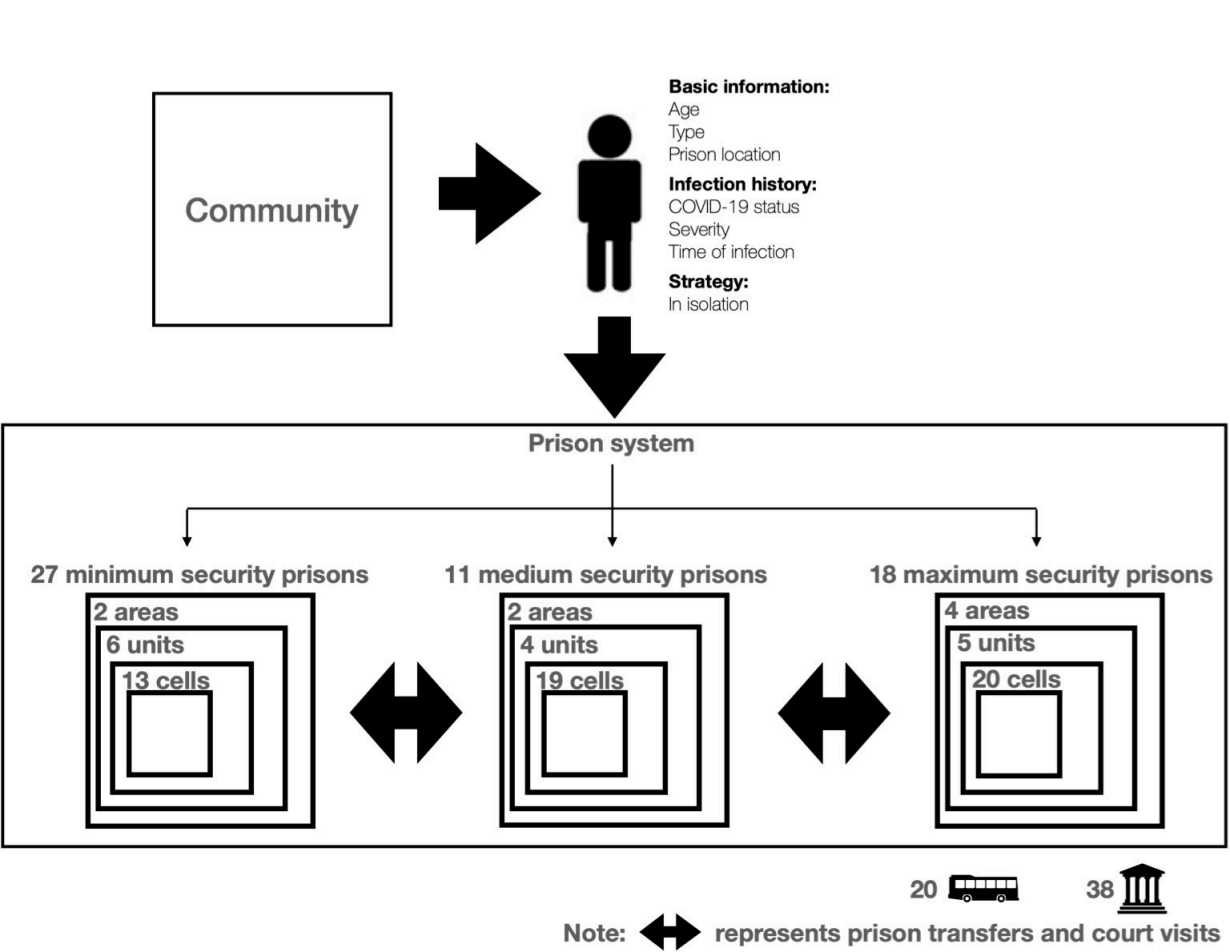
Structure of the model. The model represents the NSW prison system consisting of prisons with varying security settings. Each prison consists of areas, which consists of units, which consists of cells. The model considers the possibility transfers between prisons, as well as visits to 38 courts via 20 transfer buses.

The model simulated 13,458 inmates, 6,909 correctional staff, and 578 healthcare staff, based on population data as of December 2019. It reflects the real-world structure of the NSW prison system where each prison consists of areas, which are composed of units (or ‘wings’), which in turn are composed of cells, which house up to two individuals (Fig 1). Inmates can interact with each other if they are in the same area of the same prison, [21] and can be transferred to another prison or visit a court [21]. Inmates also interact with correctional staff during patrols, escorting of inmates, and security interventions (e.g., breaking up fights) [21]. Healthcare staff interact with correctional staff and inmates when they are delivering medical services [21]. The probabilities of interactions between individuals were estimated from data provided on the average number of contacts per day for each individual type (S14-S16 Tables and section V in S1 File). The model recorded each inmate’s location and movement between centres, to and from court, and into the community using probabilities estimated from provided inmate movement data (S2-S13 Tables and sections II-IV in S1 File). When in transit, the model records the transfer truck inmates are in and the court location they attend, allowing interaction between inmates from other prisons.

### Infection and disease progression

**Fig 2:**
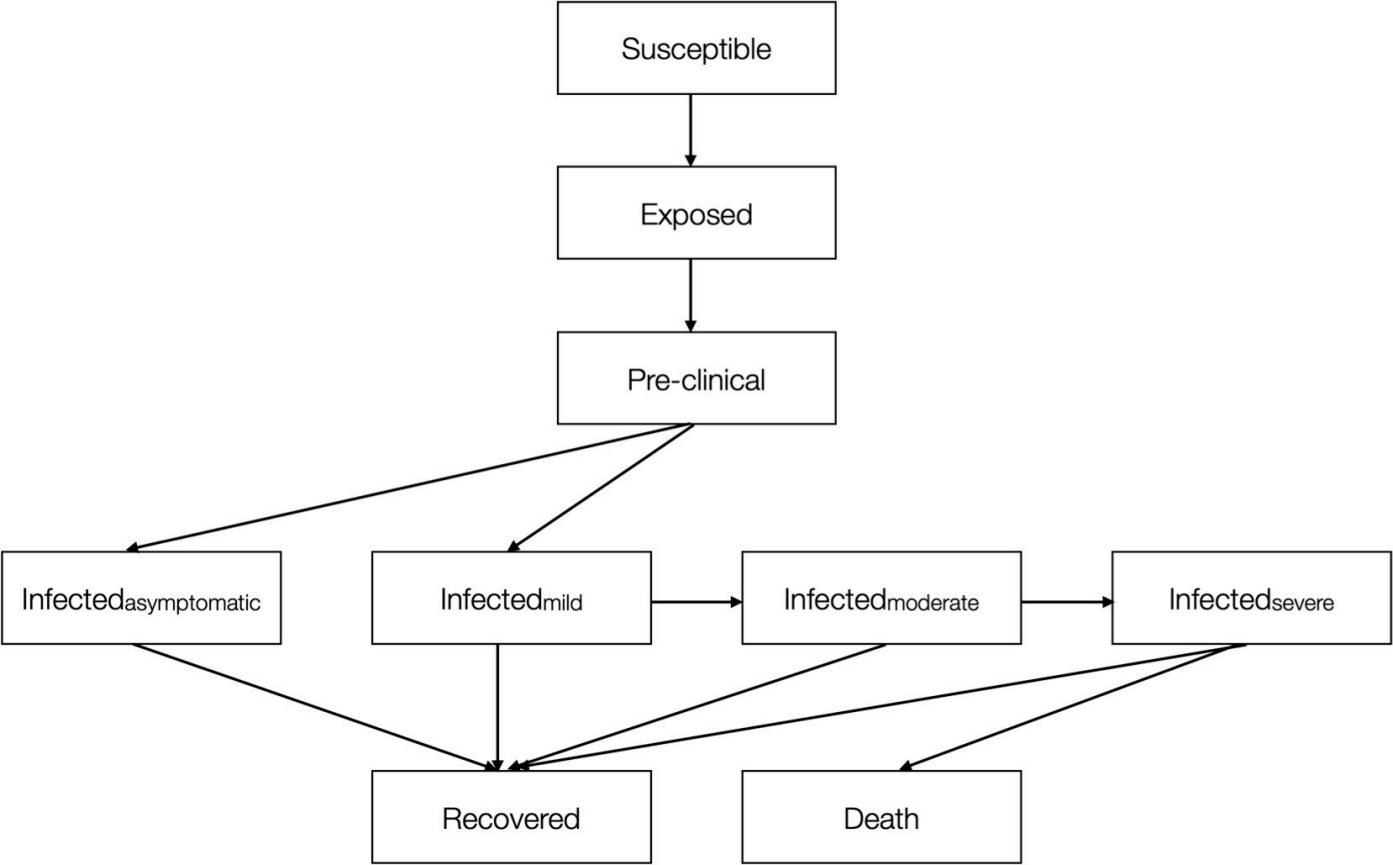
Schematic diagram of the model showing COVID-19 disease states and progression.

SARS-CoV-2 infection and COVID-19 disease progression are tracked for each simulated individual using nine disease states as shown in Fig 2 (S17 Table in S1 File). An infection probability per contact with an infectious individual and disease progression rates were obtained from published literature (Table 1; section I and VI in S1 File). Acquisition of SARS-CoV-2 occurs among those who have never been infected with SARS-Cov-2 following data specified contacts with those in the same prison area, not currently in isolation. This was then implemented as an event using a uniform probability distribution with range 0·02–0·05 for the SARS-CoV-2 alpha strain; [22] multiplied by 2 for the delta strain; and multiplied by 4 for the omicron strain (Table 1; section I in S1 File) [23]. Staff who become infected were immediately removed from the centre within 24 hours of onset of symptoms or diagnosis. As the simulation runs for only 120 days, infected individuals who recovered from infection were deemed not susceptible to reinfection.

### Model parameterization

Parameters describing new entrants, movements between prisons, and release to community were set to match the NSW inmate population within each prison using a grid search method. This resulted in a stable prisoner population for the duration of the simulations (sections VI-VII in S1 File). Age-dependent mortality rates were adjusted using the same method to match published infection fatality ratios (S18-S19 Tables and sections VI-VII in S1 File). Simulated outbreaks resulting from alpha, delta, and omicron strains were produced, with the delta strain parameters used for simulation of mitigation strategies.

### Interventions incorporated

Mitigation strategies (including a no mitigation ‘baseline’ scenario) were co-developed with correctional and health authorities to match NSW prison resources and organisational procedures. These included: personal protective equipment (PPE), quarantine on reception, isolation of proven or suspected infected cases, rapid antigen testing of staff (RAT) and inmates before transfer, prison-to-court transit restrictions, lockdown of individual prisons (i.e. no prisoner movements from centres with cases), and immunisation (see Table 2). For PPE, we modelled the use of standard and N95 masks. For each scenario, the virus entered the prison system via an infected individual (prisoner, healthcare staff or correctional staff member) on day 1.

**Table 2:** Scenarios simulated in the model.

Scenario	Description
Baseline	COVID-19 entry via an infected inmate on day 1; inmates can intermingle with other inmates in the same prison area; SARS-CoV-2 delta strain disease-related parameters applied.
Standard mask	Standard face masks in use for all inmates, correctional staff, and healthcare staff; This applies a 5% reduction in probability of onward transmission from the source; and a 67% protection of infection for the recipient [31] This scenario assumes 100% PPE compliance.
PPE + Quarantine + Isolation	Standard face masks are used by inmates everywhere including outside quarantine; new inmates are quarantined for 14 days with PCR tests at day 1 and day 12. If the PCR test returns a positive result (assuming 100% accuracy), the inmate is put into isolation for 14 days; N95 masks are used by staff in the isolation area with an 18% reduction in probability of onward transmission for the source; and an 85% protection from infection for the recipient is applied [31]. This scenario assumes 100% PPE compliance.
Entry via inmate, daily RAT	COVID-19 entry assumed to be from 1 infected inmate on day 1; correctional staff and healthcare staff are subjected to RAT testing every day before entering the prison. A pooled RAT sensitivity of 71% and a specificity of 99% was applied [32]. Prison and healthcare staff returning a positive RAT result are assumed to be sent home and subjected to PCR testing within 24 hours.
Entry via correctional staff, daily RAT	COVID-19 entry assumed to be from 1 infected correctional staff member on day 1; correctional staff and healthcare staff are subjected to RAT testing every day before entering the prison at day 1. A pooled RAT sensitivity of 71% and a specificity of 99% was applied [32]. Prison and healthcare staff returning a positive RAT result are assumed to be sent home and subjected to PCR testing within 24 hours.
Entry via healthcare staff, daily RAT	COVID-19 entry assumed to be from 1 infected healthcare staff member on day 1; correctional staff and healthcare staff are subjected to RAT testing every day before entering the prison at day 1. A pooled RAT sensitivity of 71% and a specificity of 99% was applied [32]. Prison and healthcare staff returning a positive RAT result are assumed to be sent home and subjected to PCR testing within 24 hours.
Entry via correctional staff daily, second daily RAT	COVID-19 entry assumed to be from 1 infected correctional staff member on day 1; correctional staff and healthcare staff are subject to RAT testing every second day before entering the prison. A pooled RAT sensitivity of 71% and a specificity of 99% was applied [32] Prison and healthcare staff returning a positive RAT result are assumed to be sent home and subjected to PCR testing within 24 hours.
Standard mask during transit	Standard PPE masks are used by all inmates in-transit with 100% compliance.
N95 mask during transit	N95 masks are used by all inmates in-transit with 100% compliance.
RAT pre-transit	Inmates are subject to RAT testing before being moved to another location. A pooled RAT sensitivity of 71% and a specificity of 99% was applied; [32] If the test returns a positive result, the movement is stopped;
Restrict prison transfers	Symptomatic inmates are tested using PCR with results returned the next day. If a COVID-19 positive inmate is confirmed, all prison transfers and all court visits are stopped for all inmates across the whole prison system.
Cell isolation	Symptomatic inmates are isolated in their cell for 14 days with PCR tests at day 1 and day 12; N95 masks are used by staff interacting with isolated inmates; standard PPE masks are used by isolated inmates; isolation occurs on a rolling basis while prison interactions outside isolated cells continue as normal.
Unit isolation	Symptomatic inmates are tested using PCR with results by the next day. Units with symptomatic inmates are locked down until no one is actively infected with COVID-19; N95 masks are used by staff interacting with isolated inmates; standard PPE masks are used by isolated inmates; inmates within the unit are free to move within the same unit, but there is no travel to or from another unit within the area of the centre; isolation occurs on a rolling basis while prison interactions outside isolated units continue as normal.
Area isolation	Symptomatic inmates are tested using PCR with results by the next day. Areas with symptomatic inmates are locked down until no one is actively infected with COVID-19; N95 masks are used by staff interacting with isolated inmates; standard PPE masks are used by isolated inmates; inmates within the area are free to move within the area but there is no travel to or from another area within the centre; isolation occurs on a rolling basis while prison interactions outside isolated areas continue as normal.
Prison lockdown (no delay)	Symptomatic inmates are tested using PCR with results by the next day. Prisons with symptomatic inmates with confirmed COVID-19 infection are locked down until no one is actively infected with COVID-19; N95 masks are used by staff interacting with isolated inmates; standard PPE masks are used by isolated inmates; prisons are locked on a rolling basis; inmates within the centre are free to move within the prison but there are no movements to or from other prisons (which operate as normal).
Prison lockdown (1-week delay)	Symptomatic inmates are tested using PCR with results by the next day; Prisons with symptomatic inmates with confirmed COVID-19 infection are locked down after 1 week until no one is actively infected with COVID-19; N95 masks are used by staff interacting with isolated inmates; standard PPE masks are used by isolated inmates; prisons are locked on a rolling basis; inmates within the centre are free to move within the prison but there are no movements to or from other prisons (which operate as normal).
Prison lockdown (3-week delay)	Symptomatic inmates are tested using PCR with results by the next day. Prisons with symptomatic inmates with confirmed COVID-19 infection are locked down after 3 weeks until no one is actively infected with COVID-19; N95 masks are used by staff interacting with isolated inmates; standard PPE masks are used by isolated inmates. Inmates within the centre are free to move within the prison but there are no movements to or from other prisons; prisons are locked on a rolling basis; inmates within the centre are free to move within the prison but there are no movements to or from other prisons (which operate as normal).
Prison lockdown (6-week delay)	Symptomatic inmates are tested using PCR with results by the next day; Prisons with symptomatic inmates with confirmed COVID-19 infection are locked down after 6 weeks until no one is actively infected with COVID-19; N95 masks are used by staff interacting with isolated inmates; standard PPE masks are used by isolated inmates; prisons are locked on a rolling basis; inmates within the centre are free to move within the prison but there are no movements to or from other prisons (which operate as normal).
Low coverage immunisation for inmates and staff	Assumes standard PPE is in place; 80% of the inmate population are assumed to have had a single dose and 50% to have had double dose immunisation; the same vaccination rate is applied for correctional and healthcare staff. We assumed the use of Pfizer mRNA vaccine. After the first dose, we applied a 46% reduction in onward COVID-19 transmission a 30% reduction in COVID-19 infection. After two doses, we applied a 65% reduction in onward COVID-19 transmission a 79% reduction in COVID-19 infection [33].
Low coverage immunisation for inmates and high coverage immunisation for staff	Assumes standard PPE is in place; All healthcare staff and all correctional staff are assumed to be double dose immunised; 80% of the inmate population are assumed to have had a single dose and 50% to have had double dose immunisation. We assumed the use of Pfizer mRNA vaccine. After the first dose, we applied a 46% reduction in onward COVID-19 transmission a 30% reduction in COVID-19 infection. After two doses, we applied a 65% reduction in onward COVID-19 transmission a 79% reduction in COVID-19 infection [33].
High coverage immunisation for inmates and low coverage immunisation for staff	Assumes standard PPE is in place; 80% of the inmate population are assumed to have had double dose immunisation; the same vaccination rate is applied for correctional and healthcare staff. We assumed the use of Pfizer mRNA vaccine. After the first dose, we applied a 46% reduction in onward COVID-19 transmission a 30% reduction in COVID-19 infection. After two doses, we applied a 65% reduction in onward COVID-19 transmission a 79% reduction in COVID-19 infection [33].
High coverage immunisation for inmates and staff	Assumes standard PPE is in place; All healthcare staff and all correctional staff are assumed to be double dose immunised; 80% of the inmate population are assumed to have had double dose immunisation. We assumed the use of Pfizer mRNA vaccine. After the first dose, we applied a 46% reduction in onward COVID-19 transmission a 30% reduction in COVID-19 infection. After two doses, we applied a 65% reduction in onward COVID-19 transmission a 79% reduction in COVID-19 infection [33].
High coverage immunisation for inmates and staff + Quarantine	Assumes *PPE + Quarantine + Isolation* scenario parameters in place; All healthcare staff and all correctional staff are assumed to be double dose immunised; 80% of the inmate population are assumed to have had double dose immunisation. We assumed the use of Pfizer mRNA vaccine. After the first dose, we applied a 46% reduction in onward COVID-19 transmission a 30% reduction in COVID-19 infection. After two doses, we applied a 65% reduction in onward COVID-19 transmission a 79% reduction in COVID-19 infection [33].

A two-sample Z-test with a *p*-value threshold of 0·05 was used to compare the distribution of the daily new infections of the three SARS-CoV-2 strains. A prison outbreak was defined as the occurrence of >5 infections per prison within the 120 days. A system-wide outbreak was defined as the occurrence of >2 prisons meeting the prison outbreak criteria. These definitions are conservative versions of the US CDC definitions [24]. The probability of an outbreak was estimated by counting the number of simulations meeting outbreak criteria out of 100 simulations.

### Model validation

In August and September 2021, the Metropolitan Remand and Reception Centre in NSW experienced a sustained delta variant outbreak following multiple entries of infected staff and new inmates. This was despite the implementation of several mitigation measures including PPE, quarantine, isolation, and initial vaccination rollout. We compared the actual daily prison-acquired case data among inmates to 100 simulations of the model with corresponding interventions in place. The model produced outbreaks with similar daily case rates (see section VIII, S1 Fig in S1 File).

## Results

**Fig 3:**
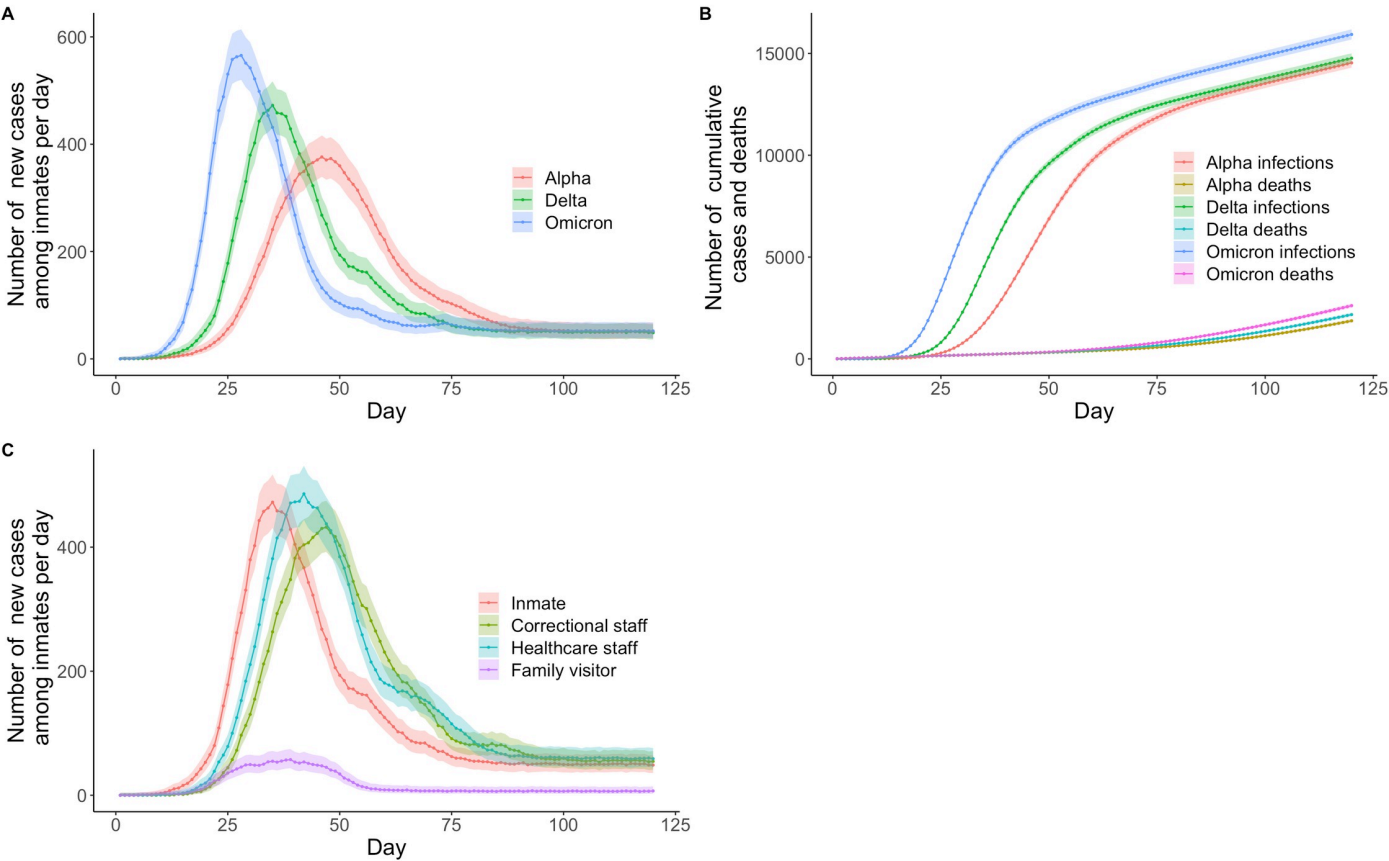
Simulation results according to SARS-CoV-2 variants and type of individual. Panel A shows a comparison of the number of new cases based on SARS-CoV-2 alpha, delta, and omicron strain transmission probabilities. Panel B shows a comparison of the number of cumulative cases and deaths using three COVID-19 variants. Panel C shows a comparison of the number of new cases given different entry points for the virus.

The three outbreak scenarios associated with the alpha, delta, and omicron strains revealed different epidemic curves, with essentially all inmates infected by day 120 and similar numbers of deaths (Fig 3A, 3B and S20-S23 Tables S1 File). The corresponding daily peak of new infections among inmates was 376 for alpha (339–416; on day 46), 472 for delta (430–517; on day 35), and 565 for omicron (519–614; on day 28). Similar outbreaks occurred when initiated by an inmate, healthcare staff, or correctional staff (Fig 3C, S24-27 Tables in S1 File) (prisoner vs correctional staff *p* = 0·87, inmate vs healthcare staff *p* = 0·33). Given these closely comparable outbreaks, all subsequent simulations were based on entry of the delta variant via an individual prisoner. Without any mitigation strategy, this scenario is referred to as the baseline scenario in which most infections were concentrated in minimum security prisons consistent with the more lenient movement restrictions placed on prisoners in these centres. There was also a sustained pattern of new daily infections among inmates in maximum security prisons, reflecting the fact that all reception prisons are designated as maximum security and continue to accept new, susceptible prison entrants from the community (S1 Video, S28 Table in S1 File). The model projected a cumulative total of 219 (43–817) healthcare staff members and 1004 (550–1862) prison officers infected by day 120 (S29 Table in S1 File).

### PPE + Quarantine + Isolation scenarios

**Fig 4:**
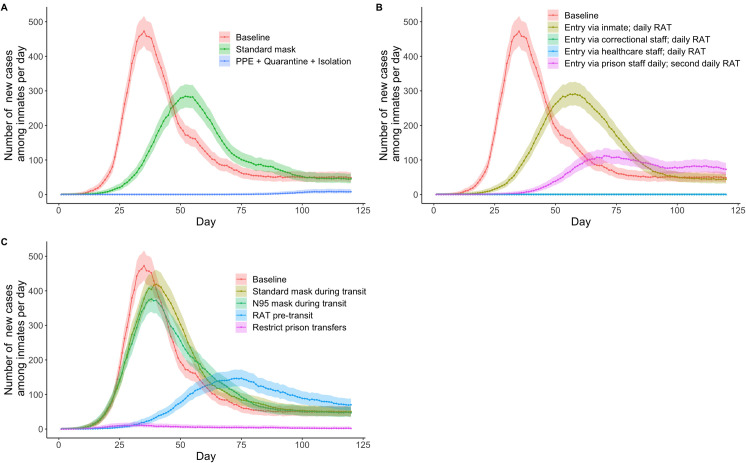
Simulation results using face mask, rapid antigen testing, and movement restriction strategies. Panel A shows a comparison of the number of new cases using various strategies involving face masks. Panel B shows a comparison of the number of new cases using different entry points for the virus and modifications in rapid antigen testing. (2 lines superimposed) Panel C shows a comparison of the number of new cases using various strategies related to movement.

In the standard mask scenario (Table 2), a peak of 284 new infections (252–319) occurred among inmates at day 52 (Fig 4A and S30 Table in S1 File). This equates to an average 21·8% (20.0%–23·5%) reduction in cumulative inmate infections compared to the baseline scenario (S31 Table in S1 File). The model projected only small outbreaks among inmates in the PPE + Quarantine + Isolation scenario [a peak of 8 (4–17) new infections at day 111 and almost 100% (99·9%–100%) reduction in cumulative infections] (Fig 4A and S32 Table in S1 File).

### RAT

Four staff RAT scenarios were modelled (Table 2) with a peak of 290 new infections (258–325) among inmates at day 56 and an average 14·8% (13·7–15·9%) reduction in cumulative inmate infections for the *Entry via inmate*, *daily RAT* scenario compared to 0 new infections among inmates for the *Entry via correctional staff*, *daily RAT* scenario and the *Entry via healthcare staff*, *daily RAT* scenario (Fig 4B and S33-S35 Tables in S1 File). Second daily testing was less effective (Fig 4B and S33 Table in S1 File).

### Transit interventions

Four scenarios evaluating the impact of control measures applied during transit of inmates within the prison system were modelled (Table 2). In the *Standard mask during transit* scenario, a peak of 418 (379–460) new inmate infections at day 40 was recorded, with no reduction in cumulative inmate infections (S36-S37 Tables in S1 File). There were fewer infections and at an earlier peak for the *N95 mask during transit* scenario [a peak of 372 (336–412) new inmate infections at day 37, no reduction in cumulative inmate infections] (S36 and S38 Tables in S1 File). In the *RAT pre-transit* scenario, there was a peak of 146 (124–172) new inmate infections at day 75 and an average 42·9% (39·7%–46.0%) reduction in cumulative inmate infections (S36 and S39 Tables in S1 File). In the *Restrict prison transfers* scenario, there was a small peak of new inmate infections and a substantial reduction in cumulative infections [11 (5–19) new inmate infections at day 34, average 96·2% (96·5%–98·4%) reduction in cumulative inmate infections] (Fig 4C and S40 Table in S1 File).

### Isolation strategies

**Fig 5:**
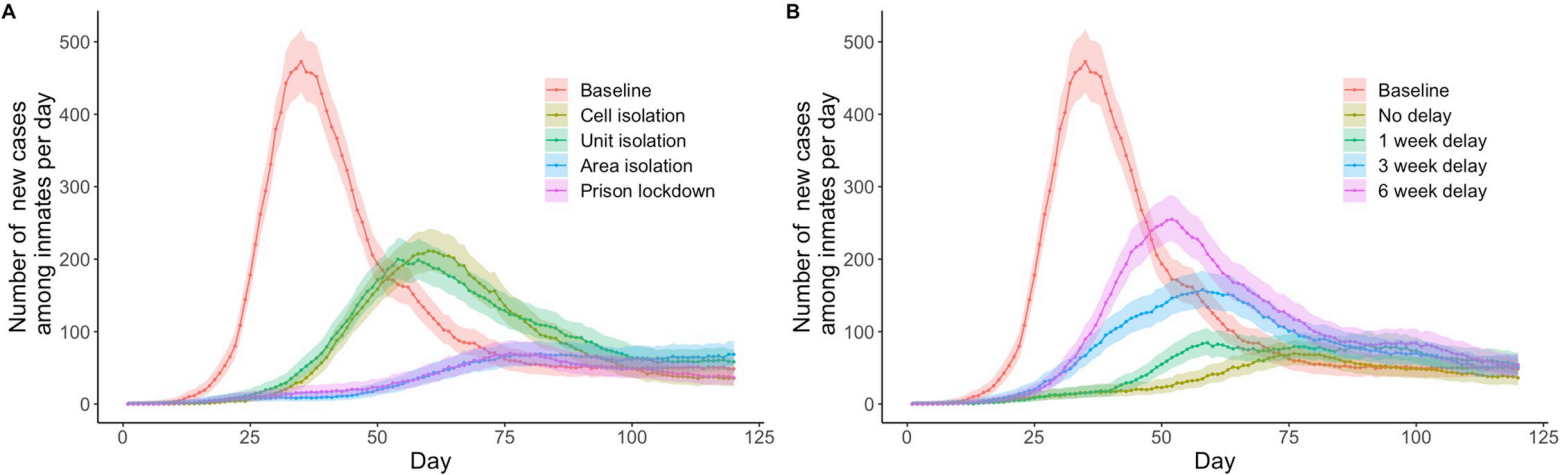
Simulation results using isolation and lockdown strategies. Simulation results according to Panel A shows a comparison of the number of new cases using various lockdown strategies. Panel B shows a comparison of the number of new cases using various delays in the implementation of prison lockdown.

Increasing the size of the population who were put in isolation due to an identified case, from cells to units to areas, progressively reduced the size of the projected outbreaks, noting that as confirmation of infection in a case is not instantaneous, larger isolation boundaries prevent transmission outside the boundary. For the *Cell isolation* scenario, a peak of 211 (183–241) new inmate infections were evident by day 60 and an average 38·3% (35·6–40·8%) reduction in cumulative inmate infections compared to a peak of 199 (173–229) new inmate infections at day 54 [33·4% (30·6–36·1%) reduction in cumulative infections] for the *Unit isolation* scenario and a peak of 68 (53–86) new inmate infections at day 119 [70·2% (65·7–74·3%) reduction in cumulative infections] for the *Area isolation* scenario (Fig 5A and S41-S44 Tables in S1 File).

### Prison lockdown

Four prison lockdown scenarios in which prisons with a confirmed case were locked down with varied timelines while the remaining prisons in the system operated normally (Table 2). In the *Prison lockdown with no delay* scenario, the model projected a peak of 69 (54–88) new inmate infections at day 76 (Fig 5b and S2 and S3 Videos), and an average 73·3% (68·6%–77·5%) reduction in cumulative infections compared to the baseline scenario (S45 Table in S1 File). Increasing the delay until lockdown beyond 1–2 weeks increased the peak size for new inmate infections and reduced the impact on cumulative infections (see Fig 5B and S46-S49 Tables in S1 File), with a 6-week delay resulting in a peak of 255 (224–288) new inmate infections at day 52 and an average 21·7% (19·7–26·5%) reduction in cumulative infections (S46-S49 Tables in S1 File).

### Immunisation strategies

**Fig 6:**
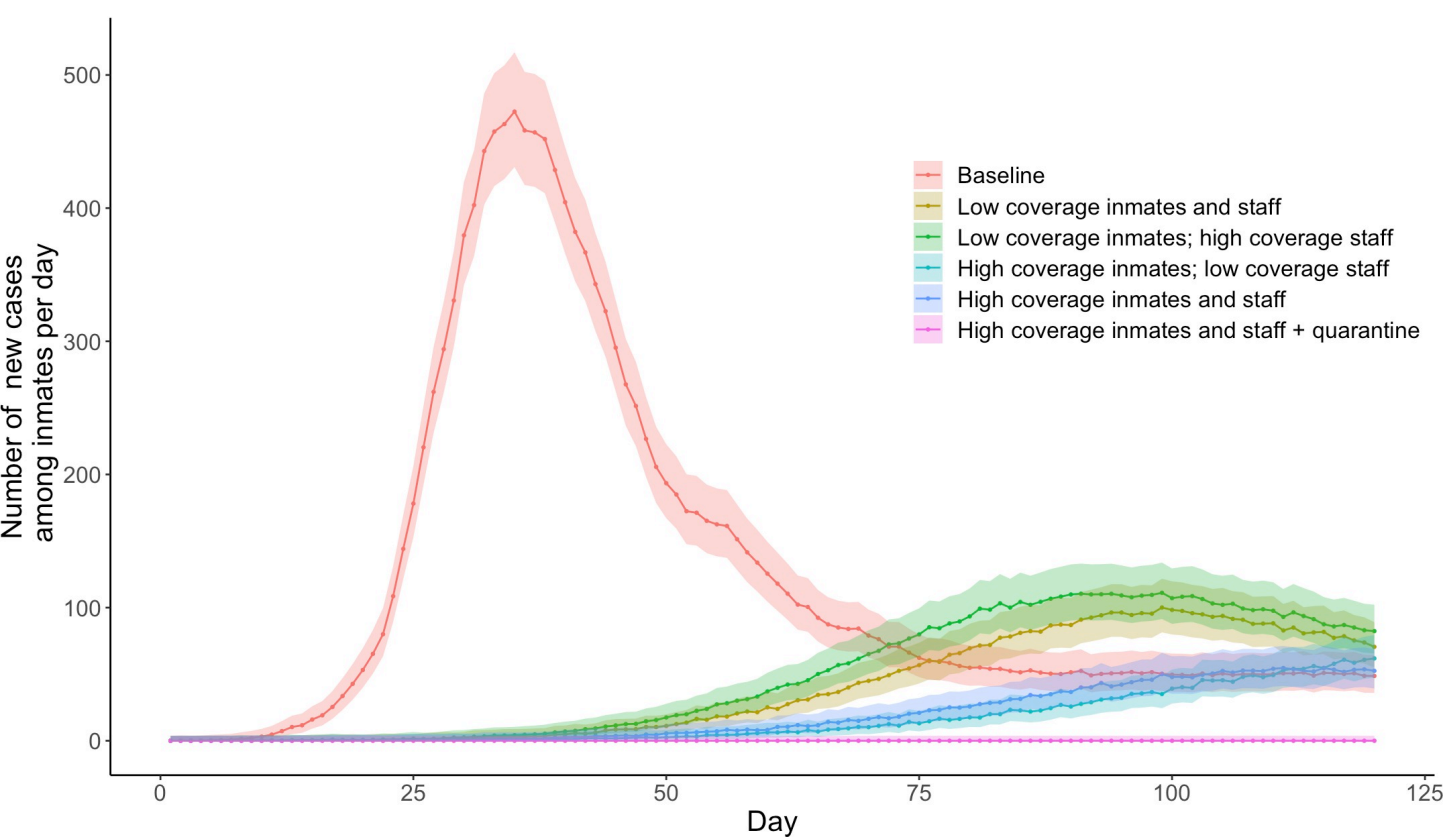
Comparison of the number of new cases using various levels of immunisation coverage.

Five scenarios involving immunisation along with standard face masks used by all inmates and staff were explored (Table 2). The magnitude of outbreaks was greatly reduced even with a low vaccination coverage (Fig 6, S50-S55 Tables in S1 File). For the *Low coverage immunisation for inmates and staff* scenario, there was a peak of 100 (81–121) new inmate infections at day 99 and an average 68·1% (72.0–63·7%) reduction in cumulative infections (S50-S51 Tables in S1 File). High vaccination coverage amongst inmates and staff substantially reduced outbreaks across the prison system but was insufficient to completely prevent outbreaks. [*High coverage immunisation for inmates and staff* scenario: projected peak 54 (41–71) new inmate infections at day 111, average 84·5% (82·6–86·2%) reduction in cumulative infections] (S50 and S54 Tables in S1 File). The addition of quarantine and isolation along with high vaccination coverage was sufficient to completely prevent outbreaks (S50 and S55 Tables in S1 File).

### Outbreak probability analysis

**Fig 7:**
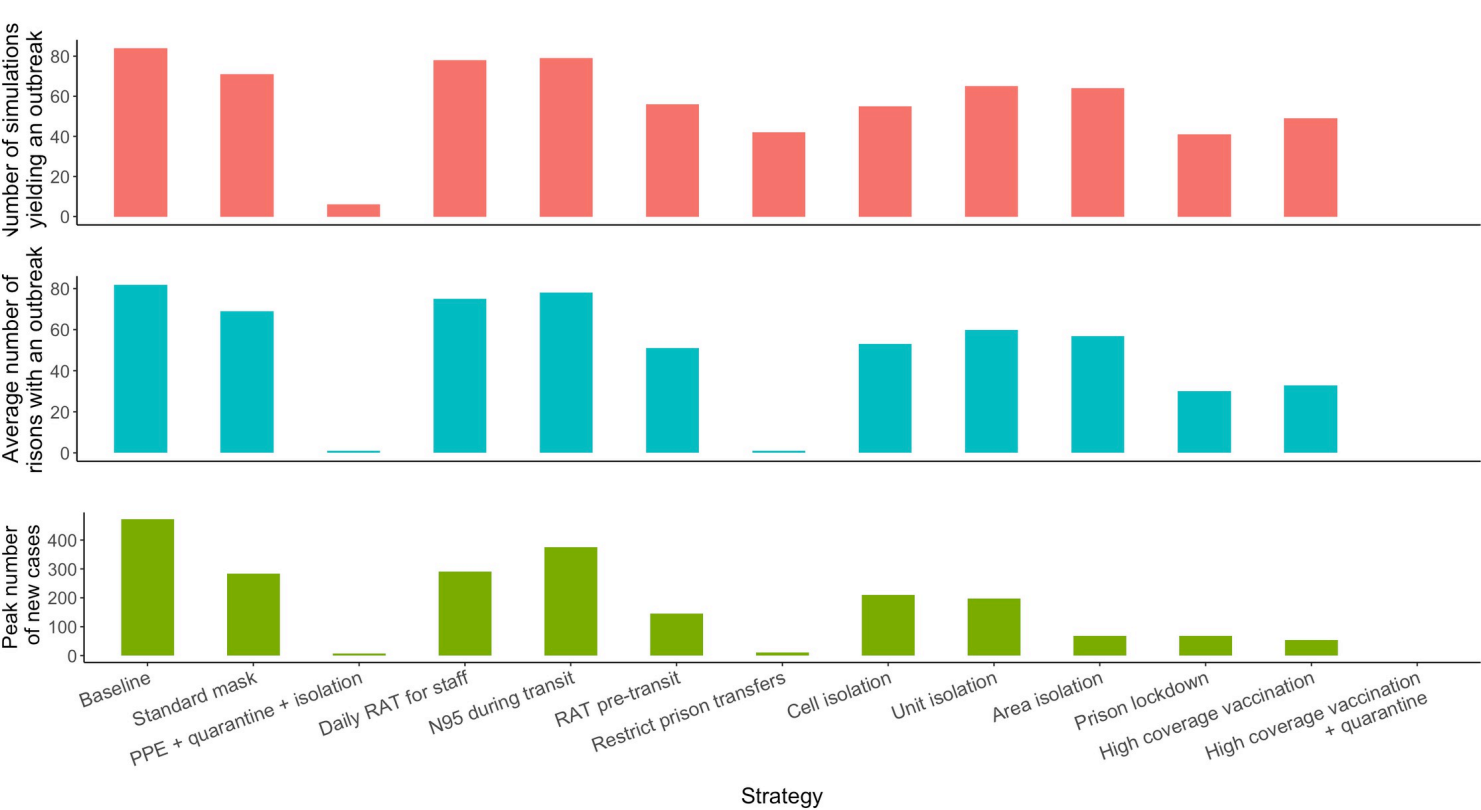
System-wide outbreak probabilities and system-wide outbreak magnitude for varied control strategies. A prison outbreak was defined as >5 infections per prison: a system/wide out 2 or more prisons that meet the prison outbreak criteria. The high coverage vaccination + quarantine strategy did not result to any secondary infections based on 1 infected new inmate over 100 simulations.

The probabilistic model allowed investigation of the number infection incursions which generate a system wide outbreak, the average number of prisons that have an outbreak, and the average number of peak inmate infections (Fig 7). As shown in Table S56 in S1 File, Implementation of the *PPE + Quarantine + Isolation* strategy markedly reduced the probability of a system wide outbreak (6 out of 100 simulations only) and limited the spread to within a single prison. The *High coverage immunisation for inmates and staff* strategy also reduced the probability of a system-wide outbreak (49 simulations), as did the *Restrict prison transfers* and *Prison lockdown (no delay)* strategy (below 50 simulations). The *Restrict prison transfers* strategy was also able to limit potential outbreaks to only one prison. The *High coverage immunisation for inmates and staff + Quarantine* strategy prevented outbreaks occurring in any of the 100 simulations of the model.

## Discussion

We developed an individual-based model that represents the whole prison system of NSW. Using this model, we utilised real-world data from correctional and health services in Australia to analyse potential COVID-19 outbreaks in a prison system. We also used this to evaluate the effectiveness of potential mitigation strategies. In the absence of control measures or a rapid outbreak response, our stochastic model projected that 100% of inmates would become infected over 120 days regardless of the SARS CoV-2 variant (alpha, delta, or omicron). The most effective individual mitigation strategies were high immunisation coverage and prompt lockdown of centres with cases which could reduce the ultimate number of cases by more than 60%. Other than immunisation, the simplest and most effective combination strategies included quarantine of inmates on entry, isolation of proven or suspected cases, and widespread use of PPE by staff and inmates.

The simulated scenarios highlight the impact of inmate movements in the spread of COVID-19 within a prison system—reiterating how critical mobility is for COVID-19 transmission [8, 14]. Strategies which restricted prison transfers, promptly locked down a centre where a case has been identified, controlling transmissions arising from entry of new inmates with infection via quarantine, and isolation of proven or suspected cases, were shown to be among the best strategies in mitigating outbreaks. This finding is concordant with a recent study showing how this approach can successfully contain an outbreak [25]. These strategies, however, require major changes in the usual custodial operations, and may markedly restrict social contact, worsen mental health, and result in violence including riots [26]. Consulting with the appropriate correctional and prison health authorities has enabled us to identify feasible and realistic mitigation strategies that can be implemented within the NSW prison system.

It is also important to note also that strategies restricting prisoner mobility were found to be time sensitive. There was a 10% system-wide reduction in the efficacy of a prison lockdown strategy if there was even a one-week delay in implementing this control measure once a COVID-19 case had been found. Moreover, delays of 6 weeks or longer were futile in preventing a major system-wide outbreak. These results were shown via an average of 100 simulations to factor in variation and uncertainty in the number of contacts and duration of contact.

Prisons are typically complex structures primarily built to ensure secure incarceration but are also commonly overcrowded at the expense of both physical and mental health [27]. Of necessity, prisons incorporate areas where congregation occurs such as shower blocks, cafeterias, and exercise yards, as well as the cells which typically have a multi-layered physical structure. This structural organization is represented in the model with cells housing either one or two inmates, organized into units or wings which share some common facilities, and which in turn are organized into areas which may typically share an exercise yard. Although these internal structures at first glance may appear to prevent the spread of COVID-19, our modelling suggests isolating a whole prison via a prompt lockdown will likely contain an outbreak within that centre, whereas isolation within internal structures is less effective. Similarly, isolation of an area is likely to be more effective than isolation of a unit or a cell–likely reflecting the fact that healthcare and correctional staff may interact with inmates across these structures, and transmissions between inmates within the smaller structures are likely to have occurred prior to, or concurrent with, an initial case detection.

Strategies such as the widespread use of PPE may not disrupt prison procedures but have only limited efficacy in outbreak control when implemented alone (even assuming face masks are correctly used 100% of the time). When combined with quarantine of all those newly incarcerated and with isolation of proven or suspected cases, PPE was highly effective in controlling outbreaks. These combined measures were comparable to prompt prison lockdowns and high coverage immunisation (where the probability of an outbreak occurring remained over 40%).

While costly, the use of daily RAT testing of all staff, was shown in the model to be very effective in preventing entry of COVID-19 into the prison system via a staff member. Interestingly, reducing the testing frequency to second daily was far less effective in preventing this portal of entry and the consequent substantive outbreaks among the prisoner population. Further, although an effective control strategy for transmission from staff members, RAT testing of all inmates prior to movement between centres only afforded a 43% reduction in cumulative infections, perhaps reflecting a larger number of daily movements of inmates (of the order of 250 movements per day in the system) and the high probability of transmission during transit.

Our study shows that high coverage immunisation of both staff and prisoners is effective in mitigating COVID-19 outbreaks. This highlights the need to include prisoners and correctional staff as priority populations in vaccination efforts against COVID-19 [28]. Regardless of the coverage, this strategy is comparable to a timely implementation of a prison lockdown strategy. Importantly however, the modelling indicates that high coverage immunisation alone is insufficient in preventing COVID-19 outbreaks. This concern may become increasingly evident if additional new variants emerge and vaccine-conferred cross-protection wanes [29]. The best outcome was achieved when a high vaccination coverage is implemented in combination with the use of PPE and quarantine and isolation.

While this model presents a detailed and sophisticated representation of potential COVID-19 outbreaks and the effectiveness of mitigation strategies in the prison system, it is important to note the limitations. First, the data utilised in this model represents the NSW prison system, which might not necessarily reflect other prison systems. However, our simulation represents common key factors present in most prison systems worldwide including prison transfers, enclosed living quarters, and interactions with staff. While this study is applied to the NSW prison system, the model is made available online under an open access license and can be modified to represent other prison systems. Second, although the outbreak size in the baseline scenario was comparable between alpha, delta and omicron strains, the scenarios were based on the COVID-19 delta variant and its parameterisation. It is also important to note that as evidence grows around the transmission rates for COVID-19, the rates may differ from those applied in this model. The comparison that between variants reported here validates the relationship between transmission rates and the epidemic curve (a higher transmission rate results to a higher number of people infected in a fewer number of days). Third, while the structure of the prison system was modelled in moderate detail, there are elements that have not been incorporated such as ventilation and random mixing, which may impact the spread of the virus. The model also omits the possibility of inmates becoming infected from non-prison entities such as civilians in the courts. Fourth, the growing impact of immunisation and prior episodes of infection on reducing morbidity and mortality have not been included in the parameterisation used here. Lastly, while the selection of interventions was done in close consultation with prison authorities, our study did not include a cost-effectiveness analysis of the implementation of the interventions considered. Incorporating financial constraints might impact how these interventions might be implemented in the real-world. Future work should consider this.

## Conclusion

While known measures to prevent and control COVID-19 outbreaks have been adopted in the general population, such measures are not necessarily feasible in many prison systems across the globe. This is due to many differences in the community and the prison setting including higher mobility and access to healthcare services. Based on the findings in this model, a range of effective mitigation strategies can be selected for deployment in prisons and similar high-risk enclosed settings in response to outbreaks of COVID-19 or other respiratory pathogens. These modelling outputs have been used to inform public health policy and practice in several Australian prison jurisdictions. By carefully representing the real-world structure of the NSW prisons, the model can also be extended to study emerging SARS CoV-2 variants of concern, as well as other similarly transmissible respiratory pathogens.

## Supporting information

S1 File A file containing all supplementary figures and tables along with supplementary information. (DOCX)

S1 Video An animated visualisation of the average number of new cases under the baseline scenario. The data shown refers to the number of new cases among inmates from representative prisons of varying security classification in NSW prisons. The y-axis represents the number of new cases while the x-axis represents the simulation time from day 1 to 120.
(MOV)

S2 Video An animated visualisation of the average number of new cases under the lockdown scenario. The data shown refers to the number of new cases among inmates from representative prisons of varying security classification in NSW prisons. The y-axis represents the number of new cases while the x-axis represents the simulation time from day 1 to 120.
(MOV)

S3 Video An animated visualisation of the number of new cases under a single simulation of the lockdown scenario. The data shown refers to the number of new cases among inmates from representative prisons of varying security classification in NSW prisons. The y-axis represents the number of new cases while the x-axis represents the simulation time from day 1 to 120.
(MOV)
