# Supplementary material for: Controlling COVID-19 outbreaks in the correctional setting: A mathematical modelling study
Source: PLoS One. 2024 May 17;19(5):e0303062. doi: 10.1371/journal.pone.0303062 (PMC11101071; doi:10.1371/journal.pone.0303062)
Supplement: S1 File — (DOCX) [file pone.0303062.s001.docx]

Controlling COVID-19 outbreaks in the correctional setting: a mathematical modelling study *Supporting Materials*

Neil Arvin Bretaña, Jisoo Amy Kwon, Luke Grant, Jennifer Galouzis, Wendy Hoey, James Blogg, Andrew R. Lloyd, Richard T. Gray

Table of contents

[Section I. Implementation of the model 1](#_Toc122437907)

[Section II. New inmate entry 2](#_Toc122437908)

[Section III. Probability of being transferred to another prison 6](#_Toc122437909)

[Section IV. Release to community 12](#_Toc122437910)

[Section V. Number of contacts 15](#_Toc122437911)

[Section VI. COVID-19 disease progression 17](#_Toc122437912)

[Section VII. Calibration 18](#_Toc122437913)

[Section VIII. Validation 19](#_Toc122437914)

[Section IX. Results 20](#_Toc122437915)

[Section X. Outbreak comparison 21](#_Toc122437916)

# Section I. Implementation of the model

The implementation of the model was written in C++ programming language (1). The model describes a typical prison setting, including security classifications (minimum, medium, and maximum) designated as individual prison locations, demographic characteristics, and COVID-19 information as described in the main manuscript. The total prisoner population was simulated based on the flux of individuals newly incarcerated from the community, released back to the community, and transferred between security classifications. Incarcerated individuals were represented as individual agents described by demographic information, infection history, and disease prevention strategy as described in the main manuscript.

The model simulated on a time scale of 1 day with individuals probabilistically experiencing up to 10 events per day. These events are related to: release to another prison location, court visit, COVID-19 infection, COVID-19 disease progression, COVID-19 related death, start of isolation, and end of isolation.

The implementation of each of these events in the model occurs via the following algorithm:

1. The model first generates an initial population. The initial prison population of 13,358 inmates, 6,909 prison staff, and 578 healthcare staff was estimated from data provided by Corrective Services NSW. None of these initial individuals are assigned to be infected with COVID-19. Parameters used for assigning the age and specific prison location of each incarcerated individual in the initial population are listed in Table S4.
2. Model simulations begin at time *t*=1. For each time step, the total prisoner population was updated by incarcerating *n* individuals from the community into the prison. The number of newly incarcerated individuals *n* was drawn using a uniform distribution from between 56 and 62. This number represents the average new incarcerations per day in NSW estimated from the number of new inmates per prison in NSW in 2019 as reported by Corrective Services NSW (5).

Newly incarcerated individuals were assigned an age, prison location, and other characteristics based on estimated probability distributions as detailed in (Table S4).

1. The model goes through every incarcerated individual and applies in-prison events.
   1. For each individual, an event probability for each event was assigned using the specified distributions, dependent on the current characteristics of the individual (discussed in detail below).
   2. Based on each event probability, the occurrence of each event was set by drawing from a binomial distribution. This dictated whether an event would be performed for each particular time step in the model.
   3. Events set to occur were then performed in a stochastic order until all occurring events are executed or a *death* or *move out of prison* event is executed, in which the individual agent is removed from the simulation.
2. Model simulation time *t* is incremented by 1.
3. The model repeats steps 2 through to 4 until maximum simulated time *T_max_* is reached.

Regarding COVID-19 transmission, a transmission event is called per simulated day for every infected individual in the following way. For each infected individual, a pool of susceptible individuals is identified given a set of conditions:

1. The individual is in the same area of the same prison location.
2. The individual is not in isolation.
3. The individual has never been infected with COVID-19

For each individual in the susceptible pool, COVID-19 infection was implemented as an event with a probability from 0.02 to 0.05 (21).

All parameter values used in the model have been normalized into probability values per day. All distributions used in the model were as implemented in the GNU scientific library (GSL)^1^.

# Section II. New inmate entry

Upon entry of a new inmate into the prison system, the model assigns this new inmate an age group, prison location, area, unit, and cell. Table S1 lists the parameters used to assign the age group of individuals in the model. These parameters were obtained using Department of Corrective Services (DCS) data by dividing the current population size by the overall population size.

Table S1. Parameters used to assign the age of model individuals.

| Age group | Distribution | Reference |
| --- | --- | --- |
| 19 and below | Fixed value (*x* = 0.02*) | Corrective Services NSW |
| 20 to 44 | Fixed value (*x* = 0.74) | Corrective Services NSW |
| 45 to 54 | Fixed value (*x* = 0.15) | Corrective Services NSW |
| 55 to 64 | Fixed value (*x* = 0.05) | Corrective Services NSW |
| 65 to 74 | Fixed value (*x* = 0.01) | Corrective Services NSW |
| 75 to 84 | Fixed value (*x* = 0.003) | Corrective Services NSW |
| 85 and above | Fixed value (*x* = 0.001) | Corrective Services NSW |

* Values have been rounded off

Table S2 lists the parameters used to assign the prison security classification of a new inmate upon entry into the prison system. These values were estimated from data provided by DCS on the number of new inmates per prison per day. Probabilities were calculated by dividing the number of inmates per prison by the total prison population.

Table S2. Parameters used to assign the prison security classification for a new inmate.

| Prison | Distribution | Reference |
| --- | --- | --- |
| Minimum security |  |  |
| Prison 1 | Fixed value (*x* = 0.04) | Corrective Services NSW |
| Prison 2 | Fixed value (*x* = 0.004) | Corrective Services NSW |
| Prison 3 | Fixed value (*x* = 0.004) | Corrective Services NSW |
| Prison 4 | Fixed value (*x* = 0.12) | Corrective Services NSW |
| Prison 5 | Fixed value (*x* = 0.014) | Corrective Services NSW |
| Prison 6 | Fixed value (*x* = 0.01) | Corrective Services NSW |
| Prison 7 | Fixed value (*x* = 0.11) | Corrective Services NSW |
| Prison 8 | Fixed value (*x* = 0.003) | Corrective Services NSW |
| Prison 9 | Fixed value (*x* = 0.03) | Corrective Services NSW |
| Prison 10 | Fixed value (*x* = 0.04) | Corrective Services NSW |
| Prison 11 | Fixed value (*x* = 0.03) | Corrective Services NSW |
| Prison 12 | Fixed value (*x* = 0.02) | Corrective Services NSW |
| Prison 13 | Fixed value (*x* = 0.01) | Corrective Services NSW |
| Prison 14 | Fixed value (*x* = 0.006) | Corrective Services NSW |
| Prison 15 | Fixed value (*x* = 0.03) | Corrective Services NSW |
| Prison 16 | Fixed value (*x* = 0.06) | Corrective Services NSW |
| Prison 17 | Fixed value (*x* = 0.04) | Corrective Services NSW |
| Prison 18 | Fixed value (*x* = 0.06) | Corrective Services NSW |
| Prison 19 | Fixed value (*x* = 0.08) | Corrective Services NSW |
| Prison 20 | Fixed value (*x* = 0.02) | Corrective Services NSW |
| Prison 21 | Fixed value (*x* = 0.03) | Corrective Services NSW |
| Prison 22 | Fixed value (*x* = 0.06) | Corrective Services NSW |
| Prison 23 | Fixed value (*x* = 0.03) | Corrective Services NSW |
| Prison 24 | Fixed value (*x* = 0.04) | Corrective Services NSW |
| Prison 25 | Fixed value (*x* = 0.06) | Corrective Services NSW |
| Prison 26 | Fixed value (*x* = 0.005) | Corrective Services NSW |
| Prison 27 | Fixed value (*x* = 0.05) | Corrective Services NSW |
| Medium security |  |  |
| Prison 1 | Fixed value (*x* = 0.19) | Corrective Services NSW |
| Prison 2 | Fixed value (*x* = 0.02) | Corrective Services NSW |
| Prison 3 | Fixed value (*x* = 0.02) | Corrective Services NSW |
| Prison 4 | Fixed value (*x* = 0.07) | Corrective Services NSW |
| Prison 5 | Fixed value (*x* = 0.09) | Corrective Services NSW |
| Prison 6 | Fixed value (*x* = 0.06) | Corrective Services NSW |
| Prison 7 | Fixed value (*x* = 0.17) | Corrective Services NSW |
| Prison 8 | Fixed value (*x* = 0.13) | Corrective Services NSW |
| Prison 9 | Fixed value (*x* = 0.04) | Corrective Services NSW |
| Prison 10 | Fixed value (*x* = 0.02) | Corrective Services NSW |
| Prison 11 | Fixed value (*x* = 0.03) | Corrective Services NSW |
| Maximum security prison |  |  |
| Prison 1 | Fixed value (*x* = 0.06) | Corrective Services NSW |
| Prison 2 | Fixed value (*x* = 0.01) | Corrective Services NSW |
| Prison 3 | Fixed value (*x* = 0.06) | Corrective Services NSW |
| Prison 4 | Fixed value (*x* = 0.06) | Corrective Services NSW |
| Prison 5 | Fixed value (*x* = 0.01) | Corrective Services NSW |
| Prison 6 | Fixed value (*x* = 0.05) | Corrective Services NSW |
| Prison 7 | Fixed value (*x* = 0.06) | Corrective Services NSW |
| Prison 8 | Fixed value (*x* = 0.01) | Corrective Services NSW |
| Prison 9 | Fixed value (*x* = 0.01) | Corrective Services NSW |
| Prison 10 | Fixed value (*x* = 0.05) | Corrective Services NSW |
| Prison 11 | Fixed value (*x* = 0.01) | Corrective Services NSW |
| Prison 12 | Fixed value (*x* = 0.15) | Corrective Services NSW |
| Prison 13 | Fixed value (*x* = 0.07) | Corrective Services NSW |
| Prison 14 | Fixed value (*x* = 0.13) | Corrective Services NSW |
| Prison 15 | Fixed value (*x* = 0.09) | Corrective Services NSW |
| Prison 16 | Fixed value (*x* = 0.05) | Corrective Services NSW |
| Prison 17 | Fixed value (*x* = 0.07) | Corrective Services NSW |
| Prison 18 | Fixed value (*x* = 0.07) | Corrective Services NSW |

Table S3 lists the parameters used to assign the prison area for new inmates, depending on their assigned security classification, upon entry into the prison system. The number of areas per prison were provided by the DCS. However, detailed data on the specific area allocation were not made available due to security reasons. For this model, we estimated an equal distribution for each prison area. This was the same for units and cells in tables S4 and S5.

Table S3. Parameters used to assign the prison area for new inmates

| Prison | Distribution |
| --- | --- |
| Minimum security prison |  |
| Area 1 | Fixed value (*x* = 0.5) |
| Area 2 | Fixed value (*x* = 0.5) |
| Medium security prison |  |
| Area 1 | Fixed value (*x* = 0.5) |
| Area 2 | Fixed value (*x* = 0.5) |
| Maximum security prison |  |
| Area 1 | Fixed value (*x* = 0.25) |
| Area 2 | Fixed value (*x* = 0.25) |
| Area 3 | Fixed value (*x* = 0.25) |
| Area 4 | Fixed value (*x* = 0.25) |

Table S4 lists the parameters used to assign the prison unit for new inmates, depending on their assigned security classification, upon entry into the prison system.

Table S4. Parameters used to assign the prison unit for new inmates

| Prison | Distribution |
| --- | --- |
| Minimum security prison |  |
| Unit 1 | Fixed value (*x* = 0.167) |
| Unit 2 | Fixed value (*x* = 0.167) |
| Unit 3 | Fixed value (*x* = 0.167) |
| Unit 4 | Fixed value (*x* = 0.167) |
| Unit 5 | Fixed value (*x* = 0.167) |
| Unit 6 | Fixed value (*x* = 0.167) |
| Medium security prison |  |
| Unit 1 | Fixed value (*x* = 0.25) |
| Unit 2 | Fixed value (*x* = 0.25) |
| Unit 3 | Fixed value (*x* = 0.25) |
| Unit 4 | Fixed value (*x* = 0.25) |
| Maximum security prison |  |
| Unit 1 | Fixed value (*x* = 0.2) |
| Unit 2 | Fixed value (*x* = 0.2) |
| Unit 3 | Fixed value (*x* = 0.2) |
| Unit 4 | Fixed value (*x* = 0.2) |
| Unit 5 | Fixed value (*x* = 0.2) |

Table S5 lists the parameters used to assign the prison cell for new inmates, depending on their assigned security classification, upon entry into the prison system.

Table S5. Parameters used to assign the prison unit for new inmates

| Prison | Distribution |
| --- | --- |
| Minimum security prison |  |
| Cell 1 | Fixed value (*x* = 0.076) |
| Cell 2 | Fixed value (*x* = 0.076) |
| Cell 3 | Fixed value (*x* = 0.076) |
| Cell 4 | Fixed value (*x* = 0.076) |
| Cell 5 | Fixed value (*x* = 0.076) |
| Cell 6 | Fixed value (*x* = 0.076) |
| Cell 7 | Fixed value (*x* = 0.076) |
| Cell 8 | Fixed value (*x* = 0.076) |
| Cell 9 | Fixed value (*x* = 0.076) |
| Cell 10 | Fixed value (*x* = 0.076) |
| Cell 11 | Fixed value (*x* = 0.076) |
| Cell 12 | Fixed value (*x* = 0.076) |
| Cell 13 | Fixed value (*x* = 0.076) |
| Medium security prison |  |
| Cell 1 | Fixed value (*x* = 0.053) |
| Cell 2 | Fixed value (*x* = 0.053) |
| Cell 3 | Fixed value (*x* = 0.053) |
| Cell 4 | Fixed value (*x* = 0.053) |
| Cell 5 | Fixed value (*x* = 0.053) |
| Cell 6 | Fixed value (*x* = 0.053) |
| Cell 7 | Fixed value (*x* = 0.053) |
| Cell 8 | Fixed value (*x* = 0.053) |
| Cell 9 | Fixed value (*x* = 0.053) |
| Cell 10 | Fixed value (*x* = 0.053) |
| Cell 11 | Fixed value (*x* = 0.053) |
| Cell 12 | Fixed value (*x* = 0.053) |
| Cell 13 | Fixed value (*x* = 0.053) |
| Cell 14 | Fixed value (*x* = 0.053) |
| Cell 15 | Fixed value (*x* = 0.053) |
| Cell 16 | Fixed value (*x* = 0.053) |
| Cell 17 | Fixed value (*x* = 0.053) |
| Cell 18 | Fixed value (*x* = 0.053) |
| Cell 19 | Fixed value (*x* = 0.053) |
| Maximum security prison |  |
| Cell 1 | Fixed value (*x* = 0.05) |
| Cell 2 | Fixed value (*x* = 0.05) |
| Cell 3 | Fixed value (*x* = 0.05) |
| Cell 4 | Fixed value (*x* = 0.05) |
| Cell 5 | Fixed value (*x* = 0.05) |
| Cell 6 | Fixed value (*x* = 0.05) |
| Cell 7 | Fixed value (*x* = 0.05) |
| Cell 8 | Fixed value (*x* = 0.05) |
| Cell 9 | Fixed value (*x* = 0.05) |
| Cell 10 | Fixed value (*x* = 0.05) |
| Cell 11 | Fixed value (*x* = 0.05) |
| Cell 12 | Fixed value (*x* = 0.05) |
| Cell 13 | Fixed value (*x* = 0.05) |
| Cell 14 | Fixed value (*x* = 0.05) |
| Cell 15 | Fixed value (*x* = 0.05) |
| Cell 16 | Fixed value (*x* = 0.05) |
| Cell 17 | Fixed value (*x* = 0.05) |
| Cell 18 | Fixed value (*x* = 0.05) |
| Cell 19 | Fixed value (*x* = 0.05) |
| Cell 20 | Fixed value (*x* = 0.05) |

# Section III. Probability of being transferred to another prison

A prison transfer event is executed by the model based on the current prison location of an inmate. Movement probabilities vary depending on the current location of an individual. These were estimated from data provided by the DCS containing the number of inmate movements between any two locations (prison to prison, and prison to community). Movements were categorised according to prison security classification. Per-day movement probabilities were calculated by dividing the number of inmate movements by the prison population in each security classification.

If a transfer event is set to occur, the inmate is placed in a one of the 20 modelled moving trucks (each with a capacity of up to a total of 7 inmates) based the transmission probability used by the model (see Main manuscript, Table 1). The following table lists the parameters used to execute a prison transfer event for an inmate.

Table S6. Parameters used execute a prison transfer event for an inmate

| Prison | Distribution | Reference |
| --- | --- | --- |
| Minimum security |  |  |
| Prison 1 | Fixed value (*x* = 0.022) | Corrective Services NSW |
| Prison 2 | Fixed value (*x* = 0.001) | Corrective Services NSW |
| Prison 3 | Fixed value (*x* = 0.001) | Corrective Services NSW |
| Prison 4 | Fixed value (*x* = 0.063) | Corrective Services NSW |
| Prison 5 | Fixed value (*x* = 0.0) | Corrective Services NSW |
| Prison 6 | Fixed value (*x* = 0.001) | Corrective Services NSW |
| Prison 7 | Fixed value (*x* = 0.059) | Corrective Services NSW |
| Prison 8 | Fixed value (*x* = 0.0) | Corrective Services NSW |
| Prison 9 | Fixed value (*x* = 0.005) | Corrective Services NSW |
| Prison 10 | Fixed value (*x* = 0.006) | Corrective Services NSW |
| Prison 11 | Fixed value (*x* = 0.005) | Corrective Services NSW |
| Prison 12 | Fixed value (*x* = 0.015) | Corrective Services NSW |
| Prison 13 | Fixed value (*x* = 0.002) | Corrective Services NSW |
| Prison 14 | Fixed value (*x* = 0.001) | Corrective Services NSW |
| Prison 15 | Fixed value (*x* = 0.005) | Corrective Services NSW |
| Prison 16 | Fixed value (*x* = 0.005) | Corrective Services NSW |
| Prison 17 | Fixed value (*x* = 0.005) | Corrective Services NSW |
| Prison 18 | Fixed value (*x* = 0.006) | Corrective Services NSW |
| Prison 19 | Fixed value (*x* = 0.009) | Corrective Services NSW |
| Prison 20 | Fixed value (*x* = 0.005) | Corrective Services NSW |
| Prison 21 | Fixed value (*x* = 0.008) | Corrective Services NSW |
| Prison 22 | Fixed value (*x* = 0.004) | Corrective Services NSW |
| Prison 23 | Fixed value (*x* = 0.019) | Corrective Services NSW |
| Prison 24 | Fixed value (*x* = 0.007) | Corrective Services NSW |
| Prison 25 | Fixed value (*x* = 0.006) | Corrective Services NSW |
| Prison 26 | Fixed value (*x* = 0.004) | Corrective Services NSW |
| Prison 27 | Fixed value (*x* = 0.008) | Corrective Services NSW |
| Medium security |  |  |
| Prison 1 | Fixed value (*x* = 0.06) | Corrective Services NSW |
| Prison 2 | Fixed value (*x* = 0.004) | Corrective Services NSW |
| Prison 3 | Fixed value (*x* = 0.002) | Corrective Services NSW |
| Prison 4 | Fixed value (*x* = 0.004) | Corrective Services NSW |
| Prison 5 | Fixed value (*x* = 0.005) | Corrective Services NSW |
| Prison 6 | Fixed value (*x* = 0.025) | Corrective Services NSW |
| Prison 7 | Fixed value (*x* = 0.025) | Corrective Services NSW |
| Prison 8 | Fixed value (*x* = 0.024) | Corrective Services NSW |
| Prison 9 | Fixed value (*x* = 0.005) | Corrective Services NSW |
| Prison 10 | Fixed value (*x* = 0.019) | Corrective Services NSW |
| Prison 11 | Fixed value (*x* = 0.013) | Corrective Services NSW |
| Maximum security prison |  |  |
| Prison 1 | Fixed value (*x* = 0.017) | Corrective Services NSW |
| Prison 2 | Fixed value (*x* = 0.001) | Corrective Services NSW |
| Prison 3 | Fixed value (*x* = 0.007) | Corrective Services NSW |
| Prison 4 | Fixed value (*x* = 0.014) | Corrective Services NSW |
| Prison 5 | Fixed value (*x* = 0.011) | Corrective Services NSW |
| Prison 6 | Fixed value (*x* = 0.027) | Corrective Services NSW |
| Prison 7 | Fixed value (*x* = 0.010) | Corrective Services NSW |
| Prison 8 | Fixed value (*x* = 0.001) | Corrective Services NSW |
| Prison 9 | Fixed value (*x* = 0.001) | Corrective Services NSW |
| Prison 10 | Fixed value (*x* = 0.001) | Corrective Services NSW |
| Prison 11 | Fixed value (*x* = 0.005) | Corrective Services NSW |
| Prison 12 | Fixed value (*x* = 0.174) | Corrective Services NSW |
| Prison 13 | Fixed value (*x* = 0.023) | Corrective Services NSW |
| Prison 14 | Fixed value (*x* = 0.112) | Corrective Services NSW |
| Prison 15 | Fixed value (*x* = 0.077) | Corrective Services NSW |
| Prison 16 | Fixed value (*x* = 0.032) | Corrective Services NSW |
| Prison 17 | Fixed value (*x* = 0.019) | Corrective Services NSW |
| Prison 18 | Fixed value (*x* = 0.020) | Corrective Services NSW |

If a prison transfer event is called, a new prison, area, unit, and cell location is assigned for the inmate of interest. Table S7 lists the parameters used to assign a new security classification for an inmate upon a transfer event, based on current security classification. The values provided in Tables S7 and S8 were calculated from the number of daily transfers between specific prison locations of varying security settings provided by the DCS.

Table S7. Parameters used to assign a new security classification upon a transfer event

| Prison | Distribution | Reference |
| --- | --- | --- |
| Minimum security → Minimum security | Fixed value (*x* = 0.571) | Corrective Services NSW |
| Minimum security → Medium security | Fixed value (*x* = 0.286) | Corrective Services NSW |
| Minimum security → Maximum security | Fixed value (*x* = 0.143) | Corrective Services NSW |
| Medium security → Minimum security | Fixed value (*x* = 0.3) | Corrective Services NSW |
| Medium security → Medium security | Fixed value (*x* = 0.4) | Corrective Services NSW |
| Medium security → Medium security | Fixed value (*x* = 0.3) | Corrective Services NSW |
| Maximum security → Minimum security | Fixed value (*x* = 0.278) | Corrective Services NSW |
| Maximum security → Medium security | Fixed value (*x* = 0.278) | Corrective Services NSW |
| Maximum security → Maximum security | Fixed value (*x* = 0.444) | Corrective Services NSW |

Table S8 lists the parameters used to assign a new prison upon a transfer event, based on the newly assigned security classification.

Table S8. Parameters used to assign a new prison upon a transfer event

| Prison | Distribution | Reference |
| --- | --- | --- |
| Minimum security |  |  |
| Prison 1 | Fixed value (*x* = 0.07) | Corrective Services NSW |
| Prison 2 | Fixed value (*x* = 0.002) | Corrective Services NSW |
| Prison 3 | Fixed value (*x* = 0.002) | Corrective Services NSW |
| Prison 4 | Fixed value (*x* = 0.233) | Corrective Services NSW |
| Prison 5 | Fixed value (*x* = 0.002) | Corrective Services NSW |
| Prison 6 | Fixed value (*x* = 0.006) | Corrective Services NSW |
| Prison 7 | Fixed value (*x* = 0.247) | Corrective Services NSW |
| Prison 8 | Fixed value (*x* = 0.002) | Corrective Services NSW |
| Prison 9 | Fixed value (*x* = 0.033) | Corrective Services NSW |
| Prison 10 | Fixed value (*x* = 0.028) | Corrective Services NSW |
| Prison 11 | Fixed value (*x* = 0.014) | Corrective Services NSW |
| Prison 12 | Fixed value (*x* = 0.028) | Corrective Services NSW |
| Prison 13 | Fixed value (*x* = 0.008) | Corrective Services NSW |
| Prison 14 | Fixed value (*x* = 0.002) | Corrective Services NSW |
| Prison 15 | Fixed value (*x* = 0.014) | Corrective Services NSW |
| Prison 16 | Fixed value (*x* = 0.022) | Corrective Services NSW |
| Prison 17 | Fixed value (*x* = 0.022) | Corrective Services NSW |
| Prison 18 | Fixed value (*x* = 0.031) | Corrective Services NSW |
| Prison 19 | Fixed value (*x* = 0.039) | Corrective Services NSW |
| Prison 20 | Fixed value (*x* = 0.016) | Corrective Services NSW |
| Prison 21 | Fixed value (*x* = 0.025) | Corrective Services NSW |
| Prison 22 | Fixed value (*x* = 0.022) | Corrective Services NSW |
| Prison 23 | Fixed value (*x* = 0.028) | Corrective Services NSW |
| Prison 24 | Fixed value (*x* = 0.030) | Corrective Services NSW |
| Prison 25 | Fixed value (*x* = 0.030) | Corrective Services NSW |
| Prison 26 | Fixed value (*x* = 0.006) | Corrective Services NSW |
| Prison 27 | Fixed value (*x* = 0.028) | Corrective Services NSW |
| Medium security |  |  |
| Prison 1 | Fixed value (*x* = 0.308) | Corrective Services NSW |
| Prison 2 | Fixed value (*x* = 0.028) | Corrective Services NSW |
| Prison 3 | Fixed value (*x* = 0.009) | Corrective Services NSW |
| Prison 4 | Fixed value (*x* = 0.032) | Corrective Services NSW |
| Prison 5 | Fixed value (*x* = 0.06) | Corrective Services NSW |
| Prison 6 | Fixed value (*x* = 0.082) | Corrective Services NSW |
| Prison 7 | Fixed value (*x* = 0.198) | Corrective Services NSW |
| Prison 8 | Fixed value (*x* = 0.115) | Corrective Services NSW |
| Prison 9 | Fixed value (*x* = 0.028) | Corrective Services NSW |
| Prison 10 | Fixed value (*x* = 0.105) | Corrective Services NSW |
| Prison 11 | Fixed value (*x* = 0.032) | Corrective Services NSW |
| Maximum security prison |  |  |
| Prison 1 | Fixed value (*x* = 0.053) | Corrective Services NSW |
| Prison 2 | Fixed value (*x* = 0.053) | Corrective Services NSW |
| Prison 3 | Fixed value (*x* = 0.053) | Corrective Services NSW |
| Prison 4 | Fixed value (*x* = 0.053) | Corrective Services NSW |
| Prison 5 | Fixed value (*x* = 0.053) | Corrective Services NSW |
| Prison 6 | Fixed value (*x* = 0.053) | Corrective Services NSW |
| Prison 7 | Fixed value (*x* = 0.053) | Corrective Services NSW |
| Prison 8 | Fixed value (*x* = 0.053) | Corrective Services NSW |
| Prison 9 | Fixed value (*x* = 0.053) | Corrective Services NSW |
| Prison 10 | Fixed value (*x* = 0.053) | Corrective Services NSW |
| Prison 11 | Fixed value (*x* = 0.053) | Corrective Services NSW |
| Prison 12 | Fixed value (*x* = 0.053) | Corrective Services NSW |
| Prison 13 | Fixed value (*x* = 0.053) | Corrective Services NSW |
| Prison 14 | Fixed value (*x* = 0.053) | Corrective Services NSW |
| Prison 15 | Fixed value (*x* = 0.053) | Corrective Services NSW |
| Prison 16 | Fixed value (*x* = 0.053) | Corrective Services NSW |
| Prison 17 | Fixed value (*x* = 0.053) | Corrective Services NSW |
| Prison 18 | Fixed value (*x* = 0.053) | Corrective Services NSW |

Table S9 lists the parameters used to assign a new prison area upon a transfer event, based on the newly assigned security classification and prison. The number of areas per prison were provided by the DCS. However, detailed data on the specific area allocation after transfers were not made available due to security reasons. For this model, we estimated an equal distribution for each prison area. This was the same for units and cells in tables S10 and S11.

Table S9. Parameters used to assign a new prison area upon a transfer event

| Prison | Distribution |
| --- | --- |
| Minimum security prison |  |
| Area 1 | Fixed value (*x* = 0.5) |
| Area 2 | Fixed value (*x* = 0.5) |
| Medium security prison |  |
| Area 1 | Fixed value (*x* = 0.5) |
| Area 2 | Fixed value (*x* = 0.5) |
| Maximum security prison |  |
| Area 1 | Fixed value (*x* = 0.25) |
| Area 2 | Fixed value (*x* = 0.25) |
| Area 3 | Fixed value (*x* = 0.25) |
| Area 4 | Fixed value (*x* = 0.25) |

Table S10 lists the parameters used to assign a new prison unit upon a transfer event, based on the newly assigned security classification and prison.

Table S10. Parameters used to assign a new prison unit upon a transfer event

| Prison | Distribution |
| --- | --- |
| Minimum security prison |  |
| Unit 1 | Fixed value (*x* = 0.167) |
| Unit 2 | Fixed value (*x* = 0.167) |
| Unit 3 | Fixed value (*x* = 0.167) |
| Unit 4 | Fixed value (*x* = 0.167) |
| Unit 5 | Fixed value (*x* = 0.167) |
| Unit 6 | Fixed value (*x* = 0.167) |
| Medium security prison |  |
| Unit 1 | Fixed value (*x* = 0.25) |
| Unit 2 | Fixed value (*x* = 0.25) |
| Unit 3 | Fixed value (*x* = 0.25) |
| Unit 4 | Fixed value (*x* = 0.25) |
| Maximum security prison |  |
| Unit 1 | Fixed value (*x* = 0.2) |
| Unit 2 | Fixed value (*x* = 0.2) |
| Unit 3 | Fixed value (*x* = 0.2) |
| Unit 4 | Fixed value (*x* = 0.2) |
| Unit 5 | Fixed value (*x* = 0.2) |

Table S11 lists the parameters used to assign a new prison cell upon a transfer event, based on the newly assigned security classification and prison

Table S11. Parameters used to assign a new prison cell upon a transfer event

| Prison | Distribution |
| --- | --- |
| Minimum security prison |  |
| Cell 1 | Fixed value (*x* = 0.076) |
| Cell 2 | Fixed value (*x* = 0.076) |
| Cell 3 | Fixed value (*x* = 0.076) |
| Cell 4 | Fixed value (*x* = 0.076) |
| Cell 5 | Fixed value (*x* = 0.076) |
| Cell 6 | Fixed value (*x* = 0.076) |
| Cell 7 | Fixed value (*x* = 0.076) |
| Cell 8 | Fixed value (*x* = 0.076) |
| Cell 9 | Fixed value (*x* = 0.076) |
| Cell 10 | Fixed value (*x* = 0.076) |
| Cell 11 | Fixed value (*x* = 0.076) |
| Cell 12 | Fixed value (*x* = 0.076) |
| Cell 13 | Fixed value (*x* = 0.076) |
| Medium security prison |  |
| Cell 1 | Fixed value (*x* = 0.053) |
| Cell 2 | Fixed value (*x* = 0.053) |
| Cell 3 | Fixed value (*x* = 0.053) |
| Cell 4 | Fixed value (*x* = 0.053) |
| Cell 5 | Fixed value (*x* = 0.053) |
| Cell 6 | Fixed value (*x* = 0.053) |
| Cell 7 | Fixed value (*x* = 0.053) |
| Cell 8 | Fixed value (*x* = 0.053) |
| Cell 9 | Fixed value (*x* = 0.053) |
| Cell 10 | Fixed value (*x* = 0.053) |
| Cell 11 | Fixed value (*x* = 0.053) |
| Cell 12 | Fixed value (*x* = 0.053) |
| Cell 13 | Fixed value (*x* = 0.053) |
| Cell 14 | Fixed value (*x* = 0.053) |
| Cell 15 | Fixed value (*x* = 0.053) |
| Cell 16 | Fixed value (*x* = 0.053) |
| Cell 17 | Fixed value (*x* = 0.053) |
| Cell 18 | Fixed value (*x* = 0.053) |
| Cell 19 | Fixed value (*x* = 0.053) |
| Maximum security prison |  |
| Cell 1 | Fixed value (*x* = 0.05) |
| Cell 2 | Fixed value (*x* = 0.05) |
| Cell 3 | Fixed value (*x* = 0.05) |
| Cell 4 | Fixed value (*x* = 0.05) |
| Cell 5 | Fixed value (*x* = 0.05) |
| Cell 6 | Fixed value (*x* = 0.05) |
| Cell 7 | Fixed value (*x* = 0.05) |
| Cell 8 | Fixed value (*x* = 0.05) |
| Cell 9 | Fixed value (*x* = 0.05) |
| Cell 10 | Fixed value (*x* = 0.05) |
| Cell 11 | Fixed value (*x* = 0.05) |
| Cell 12 | Fixed value (*x* = 0.05) |
| Cell 13 | Fixed value (*x* = 0.05) |
| Cell 14 | Fixed value (*x* = 0.05) |
| Cell 15 | Fixed value (*x* = 0.05) |
| Cell 16 | Fixed value (*x* = 0.05) |
| Cell 17 | Fixed value (*x* = 0.05) |
| Cell 18 | Fixed value (*x* = 0.05) |
| Cell 19 | Fixed value (*x* = 0.05) |
| Cell 20 | Fixed value (*x* = 0.05) |

# Section IV. Release to community

The model simulates an inmate’s release from prison depending on their current location. Table S12 lists the parameters used to execute a release event for an inmate. The values provided in Tables S12 were calculated from the number of inmate releases from each specific prison location of varying security settings provided by the DCS.

Table S12. Parameters used to release an inmate from prison to community

| Prison | Distribution | Reference |
| --- | --- | --- |
| Minimum security |  |  |
| Prison 1 | Fixed value (*x* = 0.003) | Corrective Services NSW |
| Prison 2 | Fixed value (*x* = 0.0002) | Corrective Services NSW |
| Prison 3 | Fixed value (*x* = 0.0006) | Corrective Services NSW |
| Prison 4 | Fixed value (*x* = 0.01) | Corrective Services NSW |
| Prison 5 | Fixed value (*x* = 0.0002) | Corrective Services NSW |
| Prison 6 | Fixed value (*x* = 0.0004) | Corrective Services NSW |
| Prison 7 | Fixed value (*x* = 0.014) | Corrective Services NSW |
| Prison 8 | Fixed value (*x* = 0.0002) | Corrective Services NSW |
| Prison 9 | Fixed value (*x* = 0.004) | Corrective Services NSW |
| Prison 10 | Fixed value (*x* = 0.002) | Corrective Services NSW |
| Prison 11 | Fixed value (*x* = 0.001) | Corrective Services NSW |
| Prison 12 | Fixed value (*x* = 0.003) | Corrective Services NSW |
| Prison 13 | Fixed value (*x* = 0.001) | Corrective Services NSW |
| Prison 14 | Fixed value (*x* = 0.0008) | Corrective Services NSW |
| Prison 15 | Fixed value (*x* = 0.002) | Corrective Services NSW |
| Prison 16 | Fixed value (*x* = 0.002) | Corrective Services NSW |
| Prison 17 | Fixed value (*x* = 0.001) | Corrective Services NSW |
| Prison 18 | Fixed value (*x* = 0.002) | Corrective Services NSW |
| Prison 19 | Fixed value (*x* = 0.003) | Corrective Services NSW |
| Prison 20 | Fixed value (*x* = 0.002) | Corrective Services NSW |
| Prison 21 | Fixed value (*x* = 0.0007) | Corrective Services NSW |
| Prison 22 | Fixed value (*x* = 0.002) | Corrective Services NSW |
| Prison 23 | Fixed value (*x* = 0.003) | Corrective Services NSW |
| Prison 24 | Fixed value (*x* = 0.002) | Corrective Services NSW |
| Prison 25 | Fixed value (*x* = 0.002) | Corrective Services NSW |
| Prison 26 | Fixed value (*x* = 0.0008) | Corrective Services NSW |
| Prison 27 | Fixed value (*x* = 0.003) | Corrective Services NSW |
| Medium security |  |  |
| Prison 1 | Fixed value (*x* = 0.007) | Corrective Services NSW |
| Prison 2 | Fixed value (*x* = 0.001) | Corrective Services NSW |
| Prison 3 | Fixed value (*x* = 0.001) | Corrective Services NSW |
| Prison 4 | Fixed value (*x* = 0.002) | Corrective Services NSW |
| Prison 5 | Fixed value (*x* = 0.004) | Corrective Services NSW |
| Prison 6 | Fixed value (*x* = 0.004) | Corrective Services NSW |
| Prison 7 | Fixed value (*x* = 0.009) | Corrective Services NSW |
| Prison 8 | Fixed value (*x* = 0.009) | Corrective Services NSW |
| Prison 9 | Fixed value (*x* = 0.001) | Corrective Services NSW |
| Prison 10 | Fixed value (*x* = 0.007) | Corrective Services NSW |
| Prison 11 | Fixed value (*x* = 0.002) | Corrective Services NSW |
| Maximum security prison |  |  |
| Prison 1 | Fixed value (*x* = 0.004) | Corrective Services NSW |
| Prison 2 | Fixed value (*x* = 0.00001) | Corrective Services NSW |
| Prison 3 | Fixed value (*x* = 0.001) | Corrective Services NSW |
| Prison 4 | Fixed value (*x* = 0.002) | Corrective Services NSW |
| Prison 5 | Fixed value (*x* = 0.001) | Corrective Services NSW |
| Prison 6 | Fixed value (*x* = 0.003) | Corrective Services NSW |
| Prison 7 | Fixed value (*x* = 0.004) | Corrective Services NSW |
| Prison 8 | Fixed value (*x* = 0.005) | Corrective Services NSW |
| Prison 9 | Fixed value (*x* = 0.0004) | Corrective Services NSW |
| Prison 10 | Fixed value (*x* = 0.001) | Corrective Services NSW |
| Prison 11 | Fixed value (*x* = 0.001) | Corrective Services NSW |
| Prison 12 | Fixed value (*x* = 0.02) | Corrective Services NSW |
| Prison 13 | Fixed value (*x* = 0.008) | Corrective Services NSW |
| Prison 14 | Fixed value (*x* = 0.002) | Corrective Services NSW |
| Prison 15 | Fixed value (*x* = 0.007) | Corrective Services NSW |
| Prison 16 | Fixed value (*x* = 0.01) | Corrective Services NSW |
| Prison 17 | Fixed value (*x* = 0.005) | Corrective Services NSW |
| Prison 18 | Fixed value (*x* = 0.007) | Corrective Services NSW |

The model simulates an inmate’s court visit depending on their current location. If a court visit event is set to occur, the inmate is placed in a court with other inmates visiting a court and a COVID-19 transmission event is executed based on the transmission probability used by the model (see Main manuscript, Table 1). The table below lists the parameters used to execute a court visit event for an inmate.

Table S13. Parameters used to allow an inmate for a court visit

| Prison | Distribution | Reference |
| --- | --- | --- |
| Minimum security |  |  |
| Prison 1 | Fixed value (*x* = 0.007) | Corrective Services NSW |
| Prison 2 | Fixed value (*x* = 0.0001) | Corrective Services NSW |
| Prison 3 | Fixed value (*x* = 0.002) | Corrective Services NSW |
| Prison 4 | Fixed value (*x* = 0.02) | Corrective Services NSW |
| Prison 5 | Fixed value (*x* = 0.0) | Corrective Services NSW |
| Prison 6 | Fixed value (*x* = 0.0) | Corrective Services NSW |
| Prison 7 | Fixed value (*x* = 0.048) | Corrective Services NSW |
| Prison 8 | Fixed value (*x* = 0.0006) | Corrective Services NSW |
| Prison 9 | Fixed value (*x* = 0.009) | Corrective Services NSW |
| Prison 10 | Fixed value (*x* = 0.0001) | Corrective Services NSW |
| Prison 11 | Fixed value (*x* = 0.001) | Corrective Services NSW |
| Prison 12 | Fixed value (*x* = 0.014) | Corrective Services NSW |
| Prison 13 | Fixed value (*x* = 0.0) | Corrective Services NSW |
| Prison 14 | Fixed value (*x* = 0.0) | Corrective Services NSW |
| Prison 15 | Fixed value (*x* = 0.006) | Corrective Services NSW |
| Prison 16 | Fixed value (*x* = 0.001) | Corrective Services NSW |
| Prison 17 | Fixed value (*x* = 0.0003) | Corrective Services NSW |
| Prison 18 | Fixed value (*x* = 0.011) | Corrective Services NSW |
| Prison 19 | Fixed value (*x* = 0.015) | Corrective Services NSW |
| Prison 20 | Fixed value (*x* = 0.008) | Corrective Services NSW |
| Prison 21 | Fixed value (*x* = 0.0001) | Corrective Services NSW |
| Prison 22 | Fixed value (*x* = 0.002) | Corrective Services NSW |
| Prison 23 | Fixed value (*x* = 0.021) | Corrective Services NSW |
| Prison 24 | Fixed value (*x* = 0.003) | Corrective Services NSW |
| Prison 25 | Fixed value (*x* = 0.0001) | Corrective Services NSW |
| Prison 26 | Fixed value (*x* = 0.008) | Corrective Services NSW |
| Prison 27 | Fixed value (*x* = 0.0172) | Corrective Services NSW |
| Medium security |  |  |
| Prison 1 | Fixed value (*x* = 0.019) | Corrective Services NSW |
| Prison 2 | Fixed value (*x* = 0.0002) | Corrective Services NSW |
| Prison 3 | Fixed value (*x* = 0.006) | Corrective Services NSW |
| Prison 4 | Fixed value (*x* = 0.0003) | Corrective Services NSW |
| Prison 5 | Fixed value (*x* = 0.012) | Corrective Services NSW |
| Prison 6 | Fixed value (*x* = 0.024) | Corrective Services NSW |
| Prison 7 | Fixed value (*x* = 0.06) | Corrective Services NSW |
| Prison 8 | Fixed value (*x* = 0.035) | Corrective Services NSW |
| Prison 9 | Fixed value (*x* = 0.001) | Corrective Services NSW |
| Prison 10 | Fixed value (*x* = 0.031) | Corrective Services NSW |
| Prison 11 | Fixed value (*x* = 0.027) | Corrective Services NSW |
| Maximum security prison |  |  |
| Prison 1 | Fixed value (*x* = 0.006) | Corrective Services NSW |
| Prison 2 | Fixed value (*x* = 0.001) | Corrective Services NSW |
| Prison 3 | Fixed value (*x* = 0.002) | Corrective Services NSW |
| Prison 4 | Fixed value (*x* = 0.002) | Corrective Services NSW |
| Prison 5 | Fixed value (*x* = 0.018) | Corrective Services NSW |
| Prison 6 | Fixed value (*x* = 0.044) | Corrective Services NSW |
| Prison 7 | Fixed value (*x* = 0.019) | Corrective Services NSW |
| Prison 8 | Fixed value (*x* = 0.002) | Corrective Services NSW |
| Prison 9 | Fixed value (*x* = 0.002) | Corrective Services NSW |
| Prison 10 | Fixed value (*x* = 0.002) | Corrective Services NSW |
| Prison 11 | Fixed value (*x* = 0.008) | Corrective Services NSW |
| Prison 12 | Fixed value (*x* = 0.19) | Corrective Services NSW |
| Prison 13 | Fixed value (*x* = 0.03) | Corrective Services NSW |
| Prison 14 | Fixed value (*x* = 0.12) | Corrective Services NSW |
| Prison 15 | Fixed value (*x* = 0.04) | Corrective Services NSW |
| Prison 16 | Fixed value (*x* = 0.038) | Corrective Services NSW |
| Prison 17 | Fixed value (*x* = 0.009) | Corrective Services NSW |
| Prison 18 | Fixed value (*x* = 0.041) | Corrective Services NSW |

# Section V. Number of contacts

Aside from the prison location, the susceptible cases for every transmission event is also influenced by the number of contacts per individual. Table S14 lists the number of inmates, prison staff, and healthcare staff contacts for each inmate depending on their prison location. The range of inmate contacts were estimated from the area-level prison population data across specific prisons in each security setting provided by the DCS. The number of healthcare staff contacts were derived from the number of inmates and staff that healthcare staff comes into contact with on a daily basis provided by the DCS. The number of prison staff contacts were derived from the number of inmates and staff that prison staff comes into contact with on a daily basis provided by the DCS.

Table S14. Number of inmates, prison staff, and healthcare staff contacts per inmate

|  | Distribution | Reference |
| --- | --- | --- |
| Inmate in minimum security | 15 to 195 | Corrective Services NSW |
| Inmate in medium security | 14 to 238 | Corrective Services NSW |
| Inmate in maximum security | 30 to 175 | Corrective Services NSW |
| Prison staff in minimum security | 2 | Corrective Services NSW |
| Prison staff in medium security | 1 | Corrective Services NSW |
| Prison staff in maximum security | 2 | Corrective Services NSW |
| Healthcare staff in minimum security | 1 | Corrective Services NSW |
| Healthcare staff in medium security | 1 | Corrective Services NSW |
| Healthcare staff in maximum security | 1 | Corrective Services NSW |

Table S15. Number of inmates, prison staff, and healthcare staff contacts per prison staff

|  | Distribution | Reference |
| --- | --- | --- |
| Inmate in minimum security | 5 | Corrective Services NSW |
| Inmate in medium security | 5 | Corrective Services NSW |
| Inmate in maximum security | 5 | Corrective Services NSW |
| Prison staff in minimum security | 12 | Corrective Services NSW |
| Prison staff in medium security | 14 | Corrective Services NSW |
| Prison staff in maximum security | 39 | Corrective Services NSW |
| Healthcare staff in minimum security | 0 | Corrective Services NSW |
| Healthcare staff in medium security | 0 | Corrective Services NSW |
| Healthcare staff in maximum security | 0 | Corrective Services NSW |

Table S16. Number of inmates, prison staff, and healthcare staff contacts per healthcare staff

|  | Distribution | Reference |
| --- | --- | --- |
| Inmate in minimum security | 7 | Corrective Services NSW |
| Inmate in medium security | 6 | Corrective Services NSW |
| Inmate in maximum security | 8 | Corrective Services NSW |
| Prison staff in minimum security | 0 | Corrective Services NSW |
| Prison staff in medium security | 0 | Corrective Services NSW |
| Prison staff in maximum security | 0 | Corrective Services NSW |
| Healthcare staff in minimum security | 5 | Corrective Services NSW |
| Healthcare staff in medium security | 7 | Corrective Services NSW |
| Healthcare staff in maximum security | 9 | Corrective Services NSW |

# Section VI. COVID-19 disease progression

Table S17 lists the parameters used for COVID-19 disease progression. The values presented were based on published literature as specified.

Table S17. COVID-19 disease progression parameters.

| Progression states | Distribution | Reference |
| --- | --- | --- |
| Exposed → Pre-clinical | Gamma (a=0.048, b=6.232) | ^1^ |
| Pre-clinical → Asymptomatic | Normal | ^1^ |
| 19 and below | sigma (std)=0.058906 | ^1^ |
| 20 to 44 | sigma (std)=0.054375 | ^1^ |
| 45 to 54 | sigma (std)=0.058594 | ^1^ |
| 55 to 64 | sigma (std)=0.073750 | ^1^ |
| 65 to 74 | sigma (std)=0.065781 | ^2^ |
| 75 to 84 | sigma (std)=0.063750 | ^2^ |
| 85 and above | sigma (std)=0.063750 | ^2^ |
| Pre-clinical → Mild Symptomatic | Normal | ^2^ |
| 19 and below | sigma (std)=0.058906 | ^2^ |
| 20 to 44 | sigma (std)=0.054375 | ^2^ |
| 45 to 54 | sigma (std)=0.058594 | ^2^ |
| 55 to 64 | sigma (std)=0.073750 | ^2^ |
| 65 to 74 | sigma (std)=0.065781 | ^2^ |
| 75 to 84 | sigma (std)=0.063750 | ^2^ |
| 85 and above | sigma (std)=0.063750 | ^2^ |
| Mild → Moderate | Gamma a=0.1633, b=1.0412 | ^2^ |
| Moderate → Severe | Gamma | ^2^ |
| 19 and below | a=0.0075, b=0.0145 | ^2^ |
| 20 to 44 | a=00082, b=1.913 | ^2^ |
| 45 to 54 | a=0.0035, b=9.5657 | ^2^ |
| 55 to 64 | a=0.0084, b=6.4473 | ^2^ |
| 65 to 74 | a=0.0084, b=9.1154 | ^2^ |
| 75 to 84 | a=0.0084, b=11.3455 | ^2^ |
| 85 and above | a=0.0083, b=12.0019 | ^2^ |

# Section VII. Calibration

Calibration was performed by optimising model parameters, relevant to the model output under consideration, to match observed data. We calibrated simulated prison population against prison population data from DCS, as well as COVID-19 infection fatality ratio (IFR) from a previous study^3^.

Relevant parameters were identified by observing the sensitivity of the model output based on parameter adjustments. For instance, only parameters related to inmate movement were found to affect prison population. To optimise the parameters, a grid search method was used. Relevant parameters were tuned by iteratively searching for the parameter value that yields the least difference between the mean of 10 simulation outputs and the observed data.

The optimisation algorithm used was:

1. The value of a parameter of interest is incremented.
2. The adjusted parameter value along with the existing values of other parameters are used to build a model.
3. The model output is evaluated:
   1. If the difference between the output and the observed data is minimised, the search is carried on by repeating the process from step 1.
   2. If the difference between the output and the observed data is maximised or does not change, then the previous parameter set is used. The search is carried on by optimising the next parameter.
4. The search is concluded when the difference between the output and the observed data cannot be further minimised.

Table S18 lists the simulated values in days 1, 30, 60, 90, and 120 compares to the observed prison population from the DCS. Table S19 lists the simulated values per age group against the observed IFR^3^.

Table S18. Population

| Day | Simulated | Observed | Reference |
| --- | --- | --- | --- |
| 1 | 13,156 (12,932,13,382) | 13,458 | Corrective Services NSW |
| 30 | 12,979 (12,756, 13,204) |  | Corrective Services NSW |
| 60 | 13,037 (12,814, 13,262) |  | Corrective Services NSW |
| 90 | 13,171 (12,947, 13,398) |  | Corrective Services NSW |
| 120 | 13,355 (13,130, 13,584) |  | Corrective Services NSW |

Table S19. IFR

|  | Simulated IFR | Observed IFR | Reference |
| --- | --- | --- | --- |
| 19 and below | 9.2E-6 (2.3E-6, 8.7E-5) | 4.0E-5 | ^3^ |
| 20 to 44 | 0.045 (0.002, 0.01) | 0.009 | ^3^ |
| 45 to 54 | 0.003 (0.002, 0.008) | 0.004 | ^3^ |
| 55 to 64 | 0.005 (0.003, 0.01) | 0.013 | ^3^ |
| 65 to 74 | 0.028 (0.011, 0.055) | 0.03 | ^3^ |
| 75 to 84 | 0.05 (0.022, 0.09) | 0.06 | ^3^ |
| 85 and above | 0.108 (0.053, 0.184) | 0.078 | ^3^ |

# Section VIII. Validation

For validation purposes, we aligned the number of daily cases in the first recorded COVID-19 outbreak of a major reception prison in NSW. We set the model to reflect the interventions applied in reality in this reception prison at that time, which were: the use of PPE, isolation, and quarantine of new inmates. We set the model to introduce one infected new inmate coming into the prison system over 7 days. We ran 100 simulations and compared each simulation to the observed data provided by the DCS containing number of infected inmates per day over 90 days. Figure S1 reveals matching trend lines between the model output and the observed data.


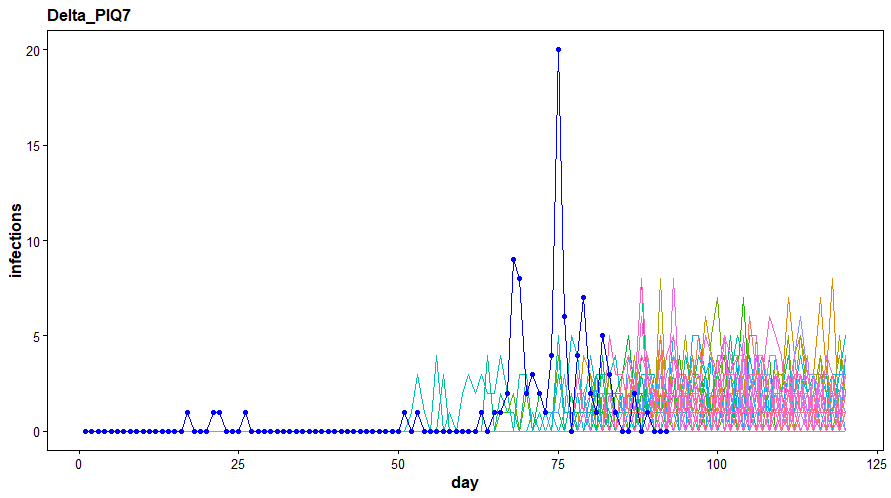


Figure S1. Comparison between 100 simulations and actual number of cases in the first COVID-19 outbreak in an NSW reception prison under implementation of PPE use, isolation, and quarantine of new prison entrants.

# Section IX. Results

This section presents a numerical summary of simulation results based on scenarios described and figures included in the main manuscript.

Table S20. Number of new cases based on SARS-CoV-2 alpha, delta, and omicron strain transmission probabilities

| Day | Alpha strain | Delta strain | Omicron strain |
| --- | --- | --- | --- |
| 1 | 0 (0, 3) | 0 (0, 3) | 0 (0, 3) |
| 30 | 132 (110, 156) | 379 (342, 419) | 542 (497, 589) |
| 60 | 222 (193, 253) | 125 (104, 149) | 71 (55, 89) |
| 90 | 57 (43, 74) | 51 (38, 67) | 54 (40, 70) |
| 120 | 50 (37, 66) | 48 (35, 64) | 51 (38, 68) |

Table S21. Number of cumulative cases and deaths based on SARS-CoV-2 alpha strain transmission probabilities

| Day | Cumulative infections | Cumulative deaths |
| --- | --- | --- |
| 1 | 0 (0, 3) | 0 (0, 0) |
| 30 | 772 (719, 829) | 185 (159, 213) |
| 60 | 9953 (9759, 10151) | 391 (353, 432) |
| 90 | 12924 (12702, 13149) | 861 (805, 921) |
| 120 | 14537 (14302, 14775) | 1868 (1785, 1955) |

Table S22. Number of cumulative cases and deaths based on SARS-CoV-2 delta strain transmission probabilities

| Day | Cumulative infections | Cumulative deaths |
| --- | --- | --- |
| 1 | 0 (0, 3) | 0 (0, 0) |
| 30 | 2295 (2202, 3391) | 186 (161, 215) |
| 60 | 11157 (10951, 11366) | 418 (378, 460) |
| 90 | 13258 (13034, 13486) | 1029 (967, 1094) |
| 120 | 14659 (14528, 15005) | 2175 (2084, 2268) |

Table S23. Number of cumulative cases and deaths based on SARS-CoV-2 omicron strain transmission probabilities

| Day | Cumulative infections | Cumulative deaths |
| --- | --- | --- |
| 1 | 0 (0, 3) | 0 (0, 0) |
| 30 | 6142 (5990, 6298) | 187 (161, 215) |
| 60 | 12570 (12351, 12791) | 471 (430, 516) |
| 90 | 14366 (14132, 14602) | 1276 (1207, 1348) |
| 120 | 15933 (15686, 16182) | 2613 (2514, 2715) |

Table S24. Number of new cases depending on COVID-19 portal of entry

| Day | Inmate | Prison staff | Healthcare staff |
| --- | --- | --- | --- |
| 1 | 0 (0, 3) | 0 (0, 3) | 0 (0, 3) |
| 30 | 379 (342, 419) | 130 (108, 154) | 210 (183, 241) |
| 60 | 117 (97, 141) | 230 (201, 262) | 180 (155, 209) |
| 90 | 52 (39, 68) | 71 (55, 89) | 64 (49, 81) |
| 120 | 48 (35, 64) | 54 (41, 71) | 58 (44, 76) |

Table S25. Number of cumulative cases and deaths based on COVID-19 entry via inmate

| Day | Cumulative infections | Cumulative deaths |
| --- | --- | --- |
| 1 | 0 (0, 3) | 0 (0, 0) |
| 30 | 2295 (2202, 3391) | 186 (161, 215) |
| 60 | 11157 (10951, 11366) | 418 (378, 460) |
| 90 | 13258 (13034, 13486) | 1029 (967, 1094) |
| 120 | 14659 (14528, 15005) | 2175 (2084, 2268) |

Table S26. Number of cumulative cases and deaths based on COVID-19 entry via prison staff

| Day | Cumulative infections | Cumulative deaths |
| --- | --- | --- |
| 1 | 0 (0, 3) | 0 (0, 0) |
| 30 | 1305 (1235, 1377) | 206 (178, 236) |
| 60 | 13906 (13676, 14139) | 426 (386, 468) |
| 90 | 15840 (15594, 16089) | 1231 (1163, 1302) |
| 120 | 17665 (17405, 17927) | 2885 (2780, 2992) |

Table S27. Number of cumulative cases and deaths based on COVID-19 entry via healthcare staff

| Day | Cumulative infections | Cumulative deaths |
| --- | --- | --- |
| 1 | 0 (0, 3) | 0 (0, 0) |
| 30 | 894 (837, 955) | 180 (154, 208) |
| 60 | 11766 (11554, 11981) | 386 (348, 426) |
| 90 | 15346 (15104, 15591) | 940 (881, 1002) |
| 120 | 17218 (16962, 17477) | 2192 (2101, 2285) |

Table S28. Number of new cases per security setting under the baseline scenario

| Day | Minimum security | Medium security | Maximum security |
| --- | --- | --- | --- |
| 1 | 0 (0, 3) | 0 (0, 3) | 0 (0, 3) |
| 30 | 187 (161, 216) | 91 (73, 112) | 100 (81, 122) |
| 60 | 45 (33, 60) | 16 (9, 27) | 63 (48, 80) |
| 90 | 2 (0, 7) | 1 (0, 5) | 47 (35, 63) |
| 120 | 1 (0, 5) | 1 (0, 5) | 46 (34, 61) |

Table S29. Case rate per 100 staff person days under the baseline scenario

| Day | Prison staff | Healthcare staff |
| --- | --- | --- |
| 1 | 0 (0, 0·052) | 0 (0, 1·2) |
| 30 | 0·112 (0·049, 0·219) | 0·286 (0·003, 1·688) |
| 60 | 0·299 (0·189, 0·448) | 1·443 (0·431, 3·329) |
| 90 | 0·052 (0·013, 0·137) | 0·651 (0·068, 2·287) |
| 120 | 0·009 (3·24E-05, 0·069) | 0·171 (7·63E-05, 1·566) |

Table S30. Number of new cases under the standard mask and PPE + Quarantine + Isolation scenarios

| Day | Baseline | Standard mask | PPE + Quarantine + Isolation |
| --- | --- | --- | --- |
| 1 | 0 (0, 3) | 0 (0, 3) | 0 (0, 3) |
| 30 | 379 (342, 419) | 49 (36, 65) | 0 (0, 3) |
| 60 | 117 (97, 141) | 235 (206, 267) | 0 (0, 3) |
| 90 | 52 (39, 68) | 72 (57, 91) | 2 (0, 7) |
| 120 | 48 (35, 64) | 45 (32, 60) | 8 (3, 16) |

Table S31. Number of cumulative cases and deaths under the standard mask scenario

| Day | Cumulative infections | Cumulative deaths |
| --- | --- | --- |
| 1 | 0 (0, 3) | 0 (0, 0) |
| 30 | 300 (267, 336) | 189 (163, 218) |
| 60 | 6431 (6274, 6590) | 387 (350, 428) |
| 90 | 10009 (9814, 10207) | 740 (688, 795) |
| 120 | 11553 (11344, 11766) | 1497 (1422, 1575) |

Table S32. Number of cumulative cases and deaths under the PPE + Quarantine + Isolation scenario

| Day | Cumulative infections | Cumulative deaths |
| --- | --- | --- |
| 1 | 0 (0, 3) | 0 (0, 0) |
| 30 | 0 (0, 3) | 0 (0, 3) |
| 60 | 0 (0, 4) | 0 (0, 3) |
| 90 | 20 (12, 31) | 0 (0, 3) |
| 120 | 226 (197, 257) | 0 (0, 4) |

Table S33. Number of new cases under various RAT scenarios

| Day | Baseline | Entry via inmate, daily RAT | Entry via prison staff, daily RAT | Entry via healthcare staff, daily RAT | Entry via prison staff daily, second daily RAT |
| --- | --- | --- | --- | --- | --- |
| 1 | 0 (0, 3) | 0 (0, 3) | 0 (0, 3) | 0 (0, 3) | 0 (0, 3) |
| 30 | 379 (342, 419) | 33 (23, 46) | 0 (0, 3) | 0 (0, 3) | 1 (0, 7) |
| 60 | 117 (97, 141) | 287 (254, 322) | 0 (0, 3) | 0 (0, 3) | 87 (70, 107) |
| 90 | 52 (39, 68) | 67 (52, 85) | 0 (0, 3) | 0 (0, 3) | 86 (69, 107) |
| 120 | 48 (35, 64) | 44 (32, 59) | 0 (0, 3) | 0 (0, 3) | 73 (57, 91) |

Table S34. Number of cumulative cases and deaths under the RAT with COVID-19 entry via inmate scenario

| Day | Cumulative infections | Cumulative deaths |
| --- | --- | --- |
| 1 | 0 (0, 3) | 0 (0, 0) |
| 30 | 215 (187, 246) | 187 (161, 216) |
| 60 | 5983 (5833, 6137) | 379 (341, 419) |
| 90 | 11083 (10878, 11292) | 710 (659, 764) |
| 120 | 12575 (12356, 12797) | 1465 (1391, 1542) |

Table S35. Number of cumulative cases and deaths under the second daily RAT with COVID-19 entry via prison staff once a day scenario

| Day | Cumulative infections | Cumulative deaths |
| --- | --- | --- |
| 1 | 0 (0, 3) | 0 (0, 0) |
| 30 | 10 (5, 19) | 183 (157, 211) |
| 60 | 955 (896, 1018) | 370 (333, 410) |
| 90 | 3984 (3862, 4110) | 595 (548, 645) |
| 120 | 6364 (6208, 6522) | 960 (900, 1022) |

Table S36. Number of new cases under various transit-related scenarios

| Day | Baseline | Standard mark during transit | N95 mask during transit | RAT pre-transit | Restrict prison transfers |
| --- | --- | --- | --- | --- | --- |
| 1 | 0 (0, 3) | 0 (0, 3) | 0 (0, 3) | 0 (0, 3) | 0 (0, 3) |
| 30 | 379 (342, 419) | 273 (242, 308) | 246 (216, 279) | 10 (5, 19) | 10 (5, 19) |
| 60 | 117 (97, 141) | 152 (128, 178) | 173 (148, 201) | 123 (102, 147) | 4 (1, 11) |
| 90 | 52 (39, 68) | 59 (45, 76) | 55 (41, 71) | 111 (91, 133) | 3 (1, 10) |
| 120 | 48 (35, 64) | 50 (37, 66) | 46 (34, 62) | 70 (54, 88) | 1 (0, 6) |

Table S37. Number of cumulative cases and deaths under the standard mask during transit scenario

| Day | Cumulative infections | Cumulative deaths |
| --- | --- | --- |
| 1 | 0 (0, 3) | 0 (0, 0) |
| 30 | 1837 (1754, 1923) | 0 (0, 3) |
| 60 | 11349 (11141, 11560) | 32 (22, 46) |
| 90 | 14070 (13839, 14305) | 391 (353, 431) |
| 120 | 15647 (15403, 15894) | 1248 (1180, 1320) |

Table S38. Number of cumulative cases and deaths under the N95 mask during transit scenario

| Day | Cumulative infections | Cumulative deaths |
| --- | --- | --- |
| 1 | 0 (0, 3) | 0 (0, 0) |
| 30 | 1837 (1754, 1923) | 0 (0, 3) |
| 60 | 10393 (10194, 10595) | 32 (22, 46) |
| 90 | 13328 (13102, 13556) | 391 (353, 431) |
| 120 | 14841 (14603, 15082) | 1248 (1180, 1320) |

Table S39. Number of cumulative cases and deaths under the RAT pre-transit scenario

| Day | Cumulative infections | Cumulative deaths |
| --- | --- | --- |
| 1 | 0 (0, 3) | 0 (0, 0) |
| 30 | 71 (56, 90) | 0 (0, 3) |
| 60 | 1902 (1817, 1989) | 2 (0, 7) |
| 90 | 5879 (5730, 6031) | 45 (33, 61) |
| 120 | 8429 (8250, 8611) | 265 (234, 299) |

Table S40. Number of cumulative cases and deaths under the restrict prison transfer scenario

| Day | Cumulative infections | Cumulative deaths |
| --- | --- | --- |
| 1 | 0 (0, 3) | 0 (0, 3) |
| 30 | 130 (109, 155) | 0 (0, 3) |
| 60 | 360 (324, 400) | 3 (0, 8) |
| 90 | 488 (445, 533) | 21 (13, 33) |
| 120 | 564 (519, 613) | 58 (44, 75) |

Table S41. Number of new cases under various isolation scenarios

| Day | Baseline | Cell isolation | Unit isolation | Area isolation | Prison lockdown |
| --- | --- | --- | --- | --- | --- |
| 1 | 0 (0, 3) | 0 (0, 3) | 0 (0, 3) | 0 (0, 3) | 0 (0, 3) |
| 30 | 379 (342, 419) | 14 (8, 24) | 23 (14, 35) | 9 (4, 17) | 11 (6, 20) |
| 60 | 117 (97, 141) | 211 (183, 241) | 192 (166, 221) | 39 (27, 53) | 41 (29, 55) |
| 90 | 52 (39, 68) | 73 (57, 92) | 91 (73, 112) | 65 (50, 83) | 54 (40, 70) |
| 120 | 48 (35, 64) | 35 (25, 49) | 58 (44, 75) | 68 (53, 86) | 36 (25, 50) |

Table S42. Number of cumulative cases and deaths under the cell isolation scenario

| Day | Cumulative infections | Cumulative deaths |
| --- | --- | --- |
| 1 | 0 (0, 3) | 0 (0, 0) |
| 30 | 94 (76, 115) | 0 (0, 3) |
| 60 | 3588 (3471, 3707) | 3 (0, 9) |
| 90 | 7738 (7567, 7913) | 83 (66, 103) |
| 120 | 9109 (8888, 9262) | 458 (417, 502) |

Table S43. Number of cumulative cases and deaths under the unit isolation scenario

| Day | Cumulative infections | Cumulative deaths |
| --- | --- | --- |
| 1 | 0 (0, 3) | 0 (0, 0) |
| 30 | 175 (150, 203) | 0 (0, 3) |
| 60 | 3904 (3783, 4029) | 3 (0, 9) |
| 90 | 7899 (7726, 8075) | 88 (70, 108) |
| 120 | 9839 (9646, 10035) | 131 (110, 156) |

Table S44. Number of cumulative cases and deaths under the area isolation scenario

| Day | Cumulative infections | Cumulative deaths |
| --- | --- | --- |
| 1 | 0 (0, 3) | 0 (0, 0) |
| 30 | 135 (113, 160) | 0 (0, 3) |
| 60 | 715 (664, 770) | 3 (0, 9) |
| 90 | 2351 (2257, 2448) | 27 (18, 40) |
| 120 | 4341 (4212, 4472) | 476 (434, 521) |

Table S45. Number of cumulative cases and deaths under the prison lockdown scenario

| Day | Cumulative infections | Cumulative deaths |
| --- | --- | --- |
| 1 | 0 (0, 3) | 0 (0, 0) |
| 30 | 128 (107, 152) | 0 (0, 3) |
| 60 | 801 (746, 858) | 3 (0, 9) |
| 90 | 2596 (2497, 2698) | 27 (18, 40) |
| 120 | 3942(3820, 4067) | 476 (434, 521) |

Table S46. Number of new cases under delayed prison lockdown scenarios

| Day | Baseline | 1-week delay | 3-week delay | 6-week delay |
| --- | --- | --- | --- | --- |
| 1 | 0 (0, 3) | 0 (0, 3) | 0 (0, 3) | 0 (0, 3) |
| 30 | 379 (342, 419) | 12 (6, 21) | 41 (29, 56) | 41 (29, 55) |
| 60 | 117 (97, 141) | 79 (63, 99) | 154 (130, 180) | 198 (172, 228) |
| 90 | 52 (39, 68) | 81 (64, 100) | 75 (59, 94) | 81 (65, 101) |
| 120 | 48 (35, 64) | 54 (40, 70) | 51 (38, 67) | 53 (40, 70) |

Table S47. Number of cumulative cases and deaths under the 1-week delayed prison lockdown scenario

| Day | Cumulative infections | Cumulative deaths |
| --- | --- | --- |
| 1 | 0 (0, 3) | 0 (0, 0) |
| 30 | 128 (106, 152) | 0 (0, 3) |
| 60 | 1389 (1317, 1464) | 2 (0, 7) |
| 90 | 3583 (3466, 3702) | 36 (25, 49) |
| 120 | 5265 (5124, 5409) | 208 (180, 238) |

Table S48. Number of cumulative cases and deaths under the 3-week delayed prison lockdown scenario

| Day | Cumulative infections | Cumulative deaths |
| --- | --- | --- |
| 1 | 0 (0, 3) | 0 (0, 0) |
| 30 | 260 (230, 294) | 0 (0, 3) |
| 60 | 3582 (3466, 3702) | 9 (4, 17) |
| 90 | 6858 (6696, 7022) | 125 (104, 149) |
| 120 | 8903 (8719, 9090) | 524 (480, 571) |

Table S49. Number of cumulative cases and deaths under the 6-week delayed prison lockdown scenario

| Day | Cumulative infections | Cumulative deaths |
| --- | --- | --- |
| 1 | 0 (0, 3) | 0 (0, 0) |
| 30 | 273 (242, 307) | 0 (0, 3) |
| 60 | 6210 (6056, 6366) | 7 (3, 15) |
| 90 | 10230 (10033, 10430) | 164 (140, 191) |
| 120 | 12334 (12117, 12554) | 767 (713, 823) |

Table S50. Number of new cases under various immunisation scenarios

| Day | Baseline | Low coverage inmates and staff | Low coverage inmates; high coverage staff | High coverage inmates; low coverage staff | High coverage inmates and staff | High coverage inmates and staff + quarantine |
| --- | --- | --- | --- | --- | --- | --- |
| 1 | 0 (0, 3) | 0 (0, 3) | 0 (0, 3) | 0 (0, 3) | 0 (0, 3) | 0 (0, 3) |
| 30 | 379 (342, 419) | 1 (0, 6) | 2 (0, 8) | 0 (0, 5) | 0 (0, 5) | 0 (0, 3) |
| 60 | 117 (97, 141) | 25 (16, 37) | 37 (26, 51) | 6 (2, 13) | 8 (3, 15) | 0 (0, 3) |
| 90 | 52 (39, 68) | 86 (69, 107) | 109 (90, 132) | 25 (16, 37) | 36 (25, 50) | 0 (0, 3) |
| 120 | 48 (35, 64) | 70 (55, 89) | 82 (65, 102) | 61 (47, 79) | 52 (39, 68) | 0 (0, 3) |

Table S51. Number of cumulative cases and deaths under the low coverage inmate and staff immunisation scenario

| Day | Cumulative infections | Cumulative deaths |
| --- | --- | --- |
| 1 | 0 (0, 3) | 0 (0, 0) |
| 30 | 14 (8, 24) | 0 (0, 3) |
| 60 | 288 (256, 323) | 181 (155, 209) |
| 90 | 2045 (1957, 2136) | 560 (514, 616) |
| 120 | 4708 (4575, 4845) | 824 (769, 833) |

Table S52. Number of cumulative cases and deaths under the low coverage inmate and high coverage staff immunisation scenario

| Day | Cumulative infections | Cumulative deaths |
| --- | --- | --- |
| 1 | 0 (0, 3) | 0 (0, 0) |
| 30 | 19 (11, 30) | 0 (0, 3) |
| 60 | 458 (417, 502) | 182 (157, 211) |
| 90 | 2820 (2717, 2926) | 574 (528, 623) |
| 120 | 5825 (5676, 5971) | 853 (797, 912) |

Table S53. Number of cumulative cases and deaths under the high coverage inmates and low coverage staff immunisation scenario

| Day | Cumulative infections | Cumulative deaths |
| --- | --- | --- |
| 1 | 0 (0, 3) | 0 (0, 0) |
| 30 | 7 (3, 15) | 0 (0, 3) |
| 60 | 82 (65, 102) | 185 (160, 214) |
| 90 | 532 (488, 579) | 579 (533, 628) |
| 120 | 1892 (1808, 1979) | 820 (765, 878) |

Table S54. Number of cumulative cases and deaths under the high coverage inmates and staff immunisation scenario

| Day | Cumulative infections | Cumulative deaths |
| --- | --- | --- |
| 1 | 0 (0, 3) | 0 (0, 0) |
| 30 | 11 (5, 19) | 0 (0, 3) |
| 60 | 138 (116, 163) | 190 (157, 219) |
| 90 | 772 (718, 828) | 588 (541, 637) |
| 120 | 2292 (2199, 2387) | 821 (766, 880) |

Table S55. Number of cumulative cases and deaths under the high coverage inmates and staff immunisation + quarantine scenario

| Day | Cumulative infections | Cumulative deaths |
| --- | --- | --- |
| 1 | 0 (0, 3) | 0 (0, 0) |
| 30 | 0 (0, 3) | 0 (0, 3) |
| 60 | 0 (0, 3) | 0 (0, 3) |
| 90 | 0 (0, 3) | 0 (0, 3) |
| 120 | 0 (0, 3) | 0 (0, 3) |

X. Outbreak comparison

Table S56. Peak number of infections, average number of prisons with outbreaks, and number of simulations resulting to an outbreak per scenario

| Strategy | Peak infections | Average number of prisons with outbreaks | Number of simulations resulting to an outbreak |
| --- | --- | --- | --- |
| Baseline | 472 | 82 | 84 |
| Standard mask | 284 | 69 | 71 |
| PPE + Quarantine + Isolation | 8 | 1 | 6 |
| Daily RAT for prison and healthcare staff | 291 | 75 | 78 |
| N95 during transit | 376 | 78 | 79 |
| RAT pre-transit | 146 | 51 | 56 |
| Restrict prison transfers | 11 | 1 | 42 |
| Cell isolation | 211 | 53 | 55 |
| Unit isolation | 198 | 60 | 65 |
| Area isolation | 68 | 57 | 64 |
| Prison lockdown | 69 | 30 | 41 |
| High coverage vaccination | 54 | 33 | 49 |
| High coverage vaccination + Quarantine | 0 | 0 | 0 |

Supplementary video 1. This video shows the average number of new cases under the baseline scenario. The data shown refers to the number of new cases among inmates from representative prisons of varying security classification in NSW prisons. The y-axis represents the number of new cases while the x-axis represents the simulation time from day 1 to 120.

Supplementary video 2. This video shows the average number of new cases under the lockdown scenario. The data shown refers to the number of new cases among inmates from representative prisons of varying security classification in NSW prisons. The y-axis represents the number of new cases while the x-axis represents the simulation time from day 1 to 120.

Supplementary video 3. This video shows the number of new cases under a single simulation of the lockdown scenario. The data shown refers to the number of new cases among inmates from representative prisons of varying security classification in NSW prisons. The y-axis represents the number of new cases while the x-axis represents the simulation time from day 1 to 120.

References
1. Galassi M, et al. GNU Scientific Library Reference Manual (3^rd^ Ed.), ISBN 0954612078

2. Davies NG, Klepac P, Liu Y, et al. Age-dependent effects in the transmission and control of COVID-19 epidemics. *Nat Med* 2020; **26**(8): 1205-11.

3. Verity R, Okell LC, Dorigatti I, et al. Estimates of the severity of coronavirus disease 2019: a model-based analysis. *Lancet Infect Dis* 2020; **20**(6): 669-77.
